# 5-aza-2′-deoxycytidine potentiates anti-tumor immunity in colorectal peritoneal metastasis by modulating ABC A9-mediated cholesterol accumulation in macrophages

**DOI:** 10.7150/thno.66420

**Published:** 2022-01-01

**Authors:** Rongchen Shi, Kun Zhao, Teng Wang, Jing Yuan, Dapeng Zhang, Wei Xiang, Jin Qian, Na Luo, Yong Zhou, Bo Tang, Chuan Li, Hongming Miao

**Affiliations:** 1 Department of Biochemistry and Molecular Biology, Third Military Medical University (Army Medical University), Chongqing 400038, China.; 2 Chongqing Weisiteng Biotech Translational Research Institute, Chongqing 400039, China.; 3 Department of General Surgery, Southwest Hospital, Third Military Medical University (Army Medical University), Chongqing 400038, China.

**Keywords:** 5Aza, CRC-PC, macrophages, ABC A9, IL-6

## Abstract

**Background:** 5-aza-2'-deoxycytidine (5Aza), a DNA methyltransferase (DNMT) inhibitor, could activate tumor adaptive immunity to inhibit tumor progression. However, the molecular mechanisms by which 5Aza regulates tumor immune microenvironment are still not fully understood.

**Methods:** The role of 5Aza in immune microenvironment of peritoneal carcinomatosis (PC) of colorectal cancer (CRC) was investigated. The effects of 5Aza on macrophage activation were studied by flow cytometry, real-time PCR, Western blotting assays, and Drug Affinity Responsive Target Stability (DARTS). The effects of 5Aza on tumor immunity were validated in stromal macrophages and T cells from CRC patients.

**Results:** 5Aza could stimulate the activation of macrophages toward an M1-like phenotype and subsequent activation of T cells in premetastatic fat tissues, and ultimately suppress CRC-PC in immune-competent mouse models. Mechanistically, 5Aza stimulated primary mouse macrophages toward to a M1-like phenotype characterized by the increase of p65 phosphorylation and IL-6 expression. Furthermore, we screened and identified ATP-binding cassette transporter A9 (ABC A9) as a binding target of 5Aza. 5Aza induced cholesterol accumulation, p65 phosphorylation and IL-6 expression in an ABC A9-dependent manner. Pharmacological inhibition of NF-κB, or genetic depletion of IL-6 abolished the antitumor effect of 5Aza in mice. In addition, the antitumor effect of 5Aza was synergistically potentiated by conventional chemotherapeutic drugs 5-Fu or OXP. Finally, we validated the reprogramming role of 5Aza in antitumor immunity in stromal macrophages and T cells from CRC patients.

**Conclusions:** Taken together, our findings showed for the first time that 5Aza suppressed CRC-PC by regulating macrophage-dependent T cell activation in premetastatic microenvironment, meanwhile uncovered a DNA methylation-independent mechanism of 5Aza in regulating ABC A9-associated cholesterol metabolism and macrophage activation.

## Introduction

As the third common malignant tumor, colorectal cancer (CRC) is a serious threat to human health 1. In addition to the hematogenous and lymphatic routes, CRC usually gives rise to transcoelomic spread (especially after surgery) and ultimately causes peritoneal carcinomatosis (PC) 2, 3. Compared with a median survival of 18.1 months in CRC patients without PC, patients with CRC-PC had a median survival of only 6.7 months, which was considered to be in the terminal phase 4. As the current primary treatment for CRC-PC, complete surgical resection and hyperthermic intraperitoneal chemotherapy (HIPEC) have improved 5-year survival outcomes in these patients by up to 40-45% 5, 6. However, the treatment of CRC-PC has stagnated in the past decade. The development of new therapies for CRC-PC is urgently needed.

In recent years, DNA methylation inhibitors in cancer therapy has achieved remarkable efficacy. 5-aza-2'-deoxycytidine (5Aza), a previously identified DNA methyltransferase (DNMT) inhibitor, has been approved by FDA for the treatment of myelodysplastic syndrome. Initially, researchers found that 5Aza can kill tumor cells by reactivating genes that had been silenced by methylation, which control cell growth 7. However, because the effects of 5Aza are varied, the underlying mechanisms are still not fully understood. As indicated by previous studies, 5Aza can activate the immune response against tumors by stimulating the expression of endogenous retroviruses or immunosurveillance related genes in tumor cell 8, 9. Therefore, DNA methylation inhibitors combined with immunotherapy have been applied in the treatment of cancers and achieved remarkable outcomes 9, 10. Stone et al. also found that 5Aza could enhance the activity of CD8^+^ T cells and inhibit tumor growth 11. However, how the tumor immune microenvironment is regulated by 5Aza is still not fully elucidated.

Macrophages, which account for the largest number of tumor interstitial cells, play important roles in regulating adaptive immunity and tumor progression. Macrophages are ideally divided into two types *in vitro*, namely, M1-like macrophages and M2-like macrophages. It is now well-established that M1-like macrophages are pro-inflammatory and anti-tumor, while M2-like macrophages are anti-inflammatory and pro-tumor 12. Studies have revealed that cholesterol metabolism is closely related to the change of macrophage polarization 13. Subfamilies of ABCA transporters, such as ABC A1 and ABC A9 are crucial for the maintenance of cellular cholesterol homeostasis 14, 15. Genetic deletion of ABC transporters could block membrane cholesterol efflux of macrophages and inhibit tumor progression 16.

We previously established a CRC-PC model in mice and found that CRC-PC mainly colonized in visceral fats and developed by suppressing adaptive immunity 17. In addition, the regulation of macrophage and T cell activity in fat tissues by high fat diet could significantly improve CRC-PC in mice 18. Given the potential role of DNA methylation in regulating immune microenvironment, we presumed that the DNA methylation inhibitor might regulate the progression of CRC-PC.

In the present study, we established CRC-PC models in mice and further investigated the functions and mechanisms of 5Aza in regulating CRC-PC. As expected, 5Aza obviously inhibited CRC-PC. However, this effect was independent of DNMT1. In contrast, 5Aza directly bound to ATP-binding cassette transporter A9 (ABC A9) to regulate cholesterol accumulation, macrophage activation and anti-tumor responses. The novel effect of 5Aza on immune regulation was finally validated in stromal macrophages and T cells from CRC patients. Our findings implicated 5Aza's great potentials in clinical treatment of CRC-PC depending on a non-canonical mechanism.

## Methods

### Cell culture

The mouse CRC cell lines MC38, CT26, and MC38G (green fluorescent protein (GFP)-tagged MC38 cells) were maintained in our laboratory. All cells were authenticated and tested for mycoplasma. Primary macrophages and all the cell lines were cultured in Dulbecco's Modified Eagle Medium (DMEM) supplemented with 10% fetal bovine serum (FBS) and 1% penicillin-streptomycin solution. Bone marrow-derived macrophages (BMDMs) were cultured in α-MEM (SH30265.01, HyClone, American) supplemented with 10% FBS, 1% penicillin-streptomycin solution, and 40 ng/mL mouse recombinant macrophage-colony stimulating factor (M-CSF). All cells were cultured at 37 °C in a humidified 5% CO_2_ atmosphere.

### Mice

Mouse studies were approved by the Institutional Animal Care and Use Committee of Third Military Medical University (Army Medical University) and were carried out according to the “Guide for the Care and Use of Laboratory Animals” published by the US National Institutes of Health (publication no.85-23, revised 1996). All C57BL/6 or BALB/c mice were provided by the Animal Center of Third Military Medical University. Nude mice were obtained from National Model Animal Resource Information Platform (#T001475, Nanjing University, China). B6.FVB-Tg (ITGAM-DTR/EGFP)34Lan/J mice were provided by the Jackson Laboratory (Stock No: 006000). This mouse has a diphtheria toxin (DT) inducible system that transiently depletes macrophages in various tissues. After DT injection, macrophages were rapidly removed from the mouse and the mouse was labeled as macrophage^ko^ in our experimental system. IL-6^KO^ mice on the C57BL/6 background were obtained with the help of Cyagen Biosciences (Guangzhou, China).

### Brdu proliferation assay

After two hours of Brdu pretreatment, cells were treated with the Phase-Flow™ FITC Brdu Kit (#370704, BioLegend). In brief, the cells were first fixed in Buffer A for 20 min and then washed with Buffer B. After using Buffer C for 10 min, add Buffer A for another 10 min. Finally, Brdu antibody was added and incubated for 30 mins before flow detection.

### Isolation of peritoneal macrophages (PMs)

The methods in this study draw on the experiences of the published articles 19, 20. Each mouse was intraperitoneally injected with 3 mL 3% mercaptoacetate solution. The ascites of mice was extracted after 72 h. The cells were lysed by the ACK lysis buffer (R1010, Solarbio, China) at room temperature for 2 min. After centrifugation, DMEM containing 10% FBS, 1% penicillin and streptomycin were used to resuspend the cells. And then cells were collected into cell culture dishes. After 2 h, adherent cells were considered as PMs.

### Isolation of bone marrow-derived macrophages (BMDMs)

The method of this study is based on the published articles 19, 20. On day 1, the mouse was sacrificed by CO_2_, and the femur and tibia were separated. The bone marrow was washed out using a 5 mL syringe. ACK lysis buffer was used to deplete red cells for 2 min at room temperature. After centrifugation, the cells were resuspended with α-MEM medium and collected into cell culture dishes. On Day 2, the suspension cells were collected and M-CSF (#576404, BioLegend) was added into culture medium. Then cells were collected again into the petri dish. Fresh α-MEM was added to the cultured cells on day 4 and day 6. On day 7, adherent cells were considered as BMDMs.

### Western blotting assays

Total proteins from macrophages were extracted, and the protein concentration was measured by BCA kit (#P0010, Beyotime, China). The protein sample volume was about 30 µg/20 µL per well, and 10% SDS-PAGE was performed. After electrophoresis, the sample was transferred to the nitrocellulose membrane. The membrane was blocked with 5% skim milk at room temperature for 2 h. Diluted primary antibody was added and incubated overnight at 4 °C. The membrane was washed with TBST for three times, and the diluted secondary antibody was added and incubated for 1 h. The signals were stimulated with Enhanced Chemiluminescence Substrate (#BG0001, Bioground, China) and detected with a Bio-Rad ChemiDoc MP System (170-8280). The primary antibody included anti-p65 (D14E12) (#8242, Cell Signaling, the dilution ratio was 1:1000), anti-phospho-p65 (Ser536) (#3033, Cell Signaling, the dilution ratio was 1:1000), anti-DNMT1 (#5032S, Cell Signaling, the dilution ratio was 1:1000), and anti-GAPDH (#60004-1-lg, Proteintech, the dilution ratio was 1:1000).

### Real-time PCR

Total RNAs were extracted using Trizole ® reagent (#30237, CWBIO) for the reverse transcription experiment. Real-time PCR was performed using the 7900HT Fast Real Time PCR system. Samples were reacted with TB Green premix kit (#RR820a, TaKaRa, Shiga, Japan) on a 96-well plate, and three wells were repeated for each sample. Relative expression was calculated with the 2(^-DDCt^) method.

### Mouse models of peritoneal carcinomatosis (PC)

MC38 or CT26 cells (1.0×10^6^ cells in 100 µL PBS) were intraperitoneally injected into 6-week-old male C57BL/6 or BALB/c mice. This study was approved by the Institutional Animal Care and Use Committee of Third Military Medical University (Army Medical University) and was carried out in accordance with the relevant guidelines.

### Establishment of DNMT1-silenced Raw264.7 cell lines

ShRNA targeting mouse DNMT1 was mediated by the PLKO.1 lentivirus packaging system provided by Hanbio Biotechnology (Shanghai, China). Each shRNA containing lentivirus was infected with mouse macrophage line Raw264,7, and shRNA SH-DNMT1-2 (hereinafter referred to as DNMT1-KD) with better efficiency was screened out for further experiments. Cells infected with empty lentivirus vectors were used as controls (NC). The establishment of stable cell lines was verified by western blotting and real-time quantitative PCR.

### Small interfering RNA (siRNA)-mediated gene silencing

We used transfection reagent (#AD600150, Zeta Life, USA) to transfect specific siRNA of ABC A9 into cells to silence their expression according to the manufacturer's protocol. Briefly, macrophages were placed in a 6-well plate with a density of 2.5 × 10^5^ cells. Transfection was performed when the cell density was 70%. 10 µL of siRNA (100 pmol/mL) and 10ul of transfection reagent were mixed at room temperature for 15 min, and then added into a 6-well plate containing 1ml of normal DMEM. After 24 h, the culture medium was removed and fresh DMEM medium was added to continue culture.

### Mouse models of subcutaneous tumor

MC38 or CT26 cells (5.0×10^6^ cells in 100 µL PBS) were subcutaneously injected into the thigh of 6-week-old male C57BL/6 or BALB/c mice. This study was approved by the Institutional Animal Care and Use Committee of Third Military Medical University (Army Medical University) and was carried out in accordance with the relevant guidelines.

### Isolation of lymphocytes and macrophages from spleens, blood, adipose or tumor tissues

Mouse spleens were placed on a 200-mesh filter and gently ground. FACS buffer (PBS with 0.5% endotoxin-free FBS, 2 mM EDTA, and 25 mM HEPES) was used to wash the filter, and cell suspensions were collected into the centrifuge tube. The cells were lysed with ACK lysis buffer at room temperature for 2 min. and then the reaction was stopped by adding equivoluminal PBS buffer. Finally, splenocytes were collected by centrifugation and resuspended with FACS buffer.

Mouse peripheral blood was collected into a blood collection tube (171001, Kang Jian, China) to prevent clotting. After treatment with ACK lysis buffer twice as described above, the blood samples were resuspended in FACS buffer.

Mouse adipose tissue was cut into small pieces and digested in RPMI 1640 medium containing 1 mg/mL collagenase IV. After digestion at 37 °C for 45 min, cells were collected and lysed in ACK lysis buffer in the same way. Finally, the adipose samples were resuspended in FACS buffer.

Mouse tumor tissue was cut into 1 mm^3^ pieces and digested in RPMI 1640 medium (containing 1 mg/mL collagenase IV, 0.1 mg/mL hyaluronidase and 0.01 mg/mL DNA enzyme). After 60 min of digestion at 37 °C, cells were collected and lysed in ACK lysis buffer as described above. Finally, the tumor samples were resuspended in FACS buffer.

### Drug affinity responsive target stability (DARTS)

The methods refer to the published articles 21. As described below, cell protein lysates were mixed with different concentrations of 5Aza (0.4 µM, 4 µM, and 40 µM) at 37 °C for 30 min without light shock. The reaction was terminated on ice after 10 minutes of action with different proportions of diluted protease (10 mg/mL). Finally, add 5X loading buffer and heat at 95 °C for 10 min to obtain the sample. Samples can be subjected to subsequent experiments by electrophoresis.

### The measurement of cholesterol content

The treated cells were added with isopropyl alcohol and then ultrasonically broken on ice. Supernatant was collected after centrifugation. Finally, the total cholesterol content detection kit (BC1985, Solarbio, China) was used to detect the content of cholesterol in cells.

### Cellular thermal shift assay (CETSA)

The methods in this study draw on the experiences of the published articles 22. In brief, peritoneal macrophages were co-incubated with different concentrations of 5Aza for 2 h. Cells were then collected and re-suspended by pre-cooled PBS containing protease inhibitors. The cells were divided into equal portions and exposed to different temperatures for three minutes. Immediately after heating, remove and incubate the tubes at room temperature for 3 min. Then snap-freeze the heat-treated cell suspensions in liquid nitrogen. Finally, the cells were freezed-thawed by liquid nitrogen twice and centrifuged at 2000 g for 20 min to collect the supernatant for subsequent experiments.

### Patient Samples

The omental fats and tumor tissues of CRC patients undergoing surgery were collected in Southwest Hospital (Chongqing China). All the participants gave written informed consent. All the patient fats and tumors were primary and untreated before surgery. The specimens were anonymized. The patient tissues were collected according to the regulations approved by the Scientific Investigation Board of the hospital.

### Immunostaining and fluorescence-activated cell sorting (FACS)

The lymphocytes and macrophages from spleens, blood, adipose or tumor tissues were incubated with antibodies at 4 °C for 45 min followed by 2 washes in FACS buffer. Then the cells were resuspended in FACS buffer for FACS analysis. The antibody included PerCP/Cy5.5 anti-mouse CD45 Antibody (#103132, BioLegend), APC anti-mouse F4/80 Antibody (#123116, BioLegend), PE/Cy7 anti-mouse CD206 (MMR) Antibody (#141704, BioLegend), Alexa Fluor700 anti-mouse CD11c Antibody (#117320, BioLegend), Alexa Fluor700 anti-mouse CD206 (MMR) Antibody (#141734, BioLegend), PE anti-mouse CD11c Antibody (#117306, BioLegend), FITC anti-mouse CD3 Antibody (#100204, BioLegend), FITC anti-mouse CD45 Antibody (#147709, BioLegend), PE anti-mouse CD3 Antibody (#100206, BioLegend), APC/Cy7 anti-mouse CD4 Antibody (#100414, BioLegend), PerCP/Cy5.5 anti-mouse CD8a Antibody (#100734, BioLegend), APC anti-mouse CD25 Antibody (#101910, BioLegend), FITC anti-mouse FOXP3 Antibody (#320012, BioLegend) and APC anti-mouse/human IFN-γ Antibody (#505810, BioLegend). Macrophages are assessed as CD45^+^F4/80^+^CD3^-^. M1-like macrophages were marked as CD45^+^F4/80^+^CD3^-^CD11c^+^CD206^-^. M2-like macrophages were assessed as CD45^+^F4/80^+^CD3^-^CD11c^-^CD206^+^. T cells were assessed as DAPI^-^CD45^+^CD3^+^CD45R^-^. B cells were assessed as DAPI^-^CD45^+^CD3^-^CD45R^+^. CD4^+^ T cells were marked as DAPI^-^CD45^+^CD3^+^CD45R^-^CD8^-^CD4^+^. CD8^+^ T cells were assessed as DAPI^-^CD45^+^CD3^+^CD45R^-^CD8^+^CD4^-^. IFN-γ^+^ T cells were assessed as CD45^+^CD3^+^IFN-γ^+^. IFN-γ^+^CD4^+^ T cells were marked as CD45^+^CD3^+^CD8^-^CD4^+^ IFN-γ^+^. CD8^+^ T cells were assessed as CD45^+^CD3^+^CD8^+^CD4^-^ IFN-γ^+^. Tregs were assessed as CD45^+^CD3^-^CD4^+^CD25^+^FOXP3^+^.

### *In vitro* phagocytosis assays

PMs (5.0×10^5^/ well) were plated in 6-well tissue culture plate and cultured in serum-free medium for 2 h. And then 2.0 × 10^6^ MC38G cells were added. Cells were collected after co-culture at 37 °C for 4 h. PMs were stained with PerCP/Cy5.5-coupled anti-CD45 and APC-coupled anti-F4/80 antibodies, and detected by flow cytometry. A total of 40000 cells from each sample were analyzed. PMs containing GFP are considered as being phagocytosing.

### Statistical analysis

Statistical analyses were carried out using GraphPad Prism 6 (GraphPad Software, Inc.). All the data were shown as the mean ± s.e.m, and were analyzed with Student's t test or Gehan-Breslow-Wilcoxon test. For each parameter of all data presented, n.s. means not significant, * means P < 0.05, ** means P < 0.01 and *** means P < 0.005.

## Results

### 5Aza suppresses the progression of CRC-PC depending on macrophages and lymphocytes

**Figure 1:**
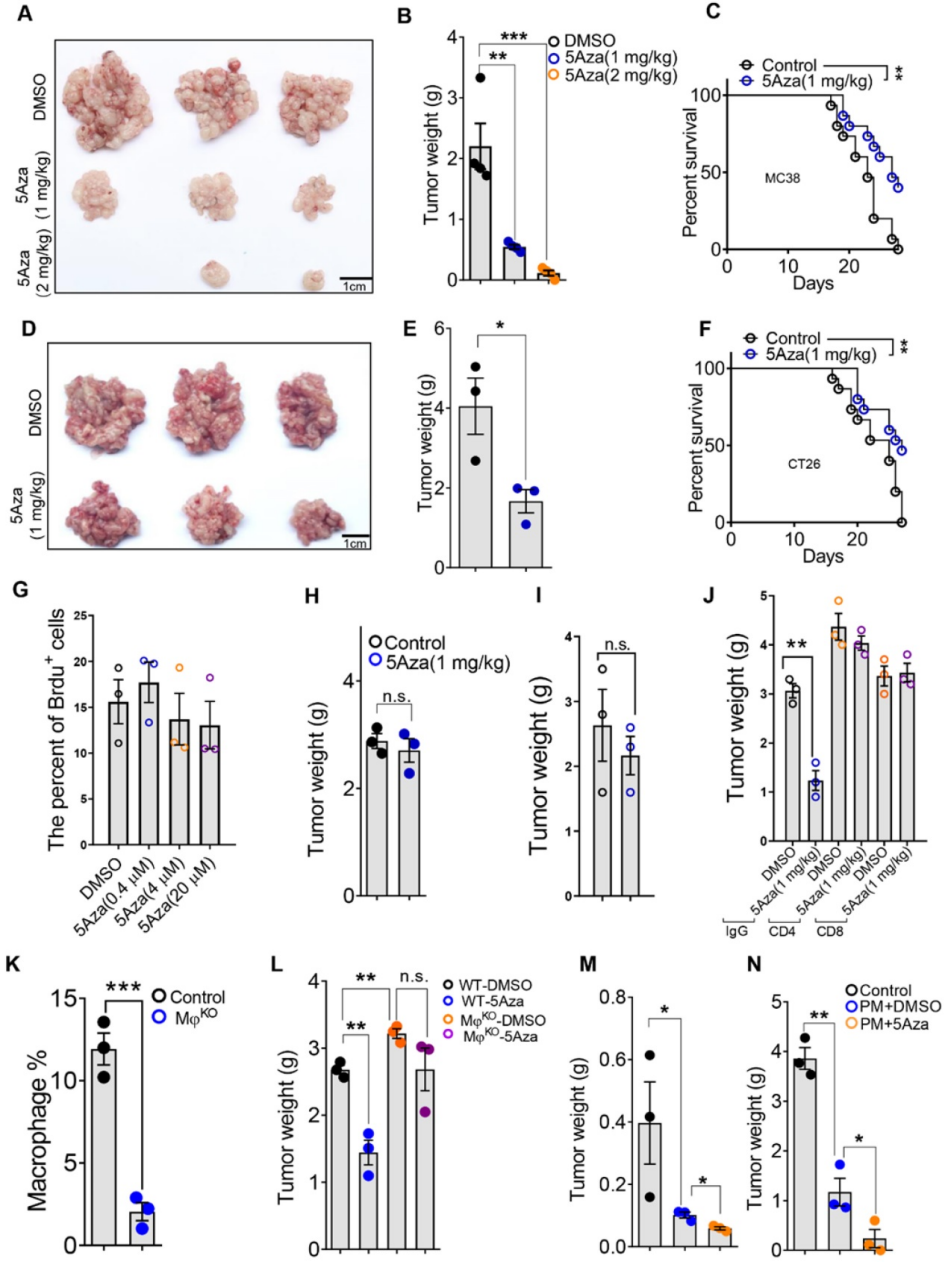
** 5Aza suppresses the progression of CRC-PC depending on macrophages and lymphocytes. A,** Tumor nodes of MC38 cells from the mice treated with DMSO or 5Aza. Six-week-old male mice were intraperitoneally injected with MC38 cells (1.0×10^6^ cells in 100 µL PBS for each mouse) and immediately treated with DMSO or 5Aza on days 0, 2, and 4. On day 14, the tumor nodes on visceral fat were displayed. **B,** Weight of the tumor nodes described in A (n = 3). **C,** The survival of tumor-seeded mice treated with DMSO or 5Aza. Tumor-seeded models were established as described in A. This experiment was repeated three times (n = 15). **D,** Tumor nodes of CT26 cells from the mice treated with DMSO or 5Aza. Tumor-seeded models were established as described in A. **E,** Weight of the tumor nodes described in D (n = 3). **F,** The survival of tumor-seeded mice described in D. This experiment was repeated three times (n = 15). **G,** Brdu proliferation assay of MC38 cells treated with different doses of 5Aza (n = 3). **H,** Weight of the tumor nodes from nude mice. Tumor-seeded models were established as described in A on day 0 and the tumor nodules were observed on day 14 (n = 3). **I,** Weight of the tumor nodes from Rag2 mice. Tumor-seeded models were established as described in A on day 0 and the tumor nodules were observed on day 14 (n = 3). **J,** Weight of the tumor nodes. Tumor-seeded models were established as described in A on day 0 and immediately treated with IgG, anti-CD4, or anti-CD8 antibody on days 0, 2, and 4 (n = 3). **K,** The percentage of total macrophages in epididymal fats. Six-week-old male macrophages^ko^ mice were intraperitoneally injected with diphtheria toxin (100 ng in 100 µL PBS for per mouse) on day 0 and were sacrificed for the analysis of total macrophages in the epididymal fats on day 1. Macrophages were marked by CD45^+^F4/80^+^ (n = 3). **L.** Weight of the tumor nodes from macrophages^ko^ mice. Tumor-seeded models were established as described in A. Tumor nodes were observed on day 14 (n = 3). **M and N,** The weight of the tumor nodes. Six-week-old male mice were subcutaneously (M) or intraperitoneally (N) injected with 5 or 1×10^6^ MC38 cells, and together with 1 or 0.2×10^5^ peritoneal macrophage after treated with DMSO or 5Aza for 48 h. Representative result was shown (n = 3). All the data were shown as the mean ± s.e.m. The data in B, E, and G-L were analyzed with Student's t test. The data in C and F were analyzed with Gehan-Breslow-Wilcoxon test. Scale bars, 1 cm (n.s., not significant, *P < 0.05, **P < 0.01, and ***P < 0.005).

To determine the role of 5Aza in CRC-PC progression, we established mouse CRC-PC models by employing colorectal cancer cell lines MC38 and CT26 (Figure S1A). We revealed that 5Aza obviously inhibited the growth of MC38 tumors in a dose dependent manner (Figure 1A-B). Importantly, the survival of mice with peritoneal metastasis was markedly improved by 5Aza treatment (Figure 1C). These antitumor effects of the 5Aza were also confirmed in another peritoneal metastasis model established with CT26 cells (Figure 1D-F).

We next explored whether 5Aza directly affected cancer cells or regulated tumor microenvironment. *In vitro* assays showed that 5Aza did not affect the viability of MC38 cells (Figure 1G and Figure S1B). Neither did 5Aza affect the apoptosis (Figure S1C), cell cycle progression (Figure S1D), or migration (Figure S1E). And the inhibitory effect of 5Aza on CRC-PC disappeared in nude mice (Figure 1H and Figure S2A). These results indicated that 5Aza suppressed CRC-PC progression through the immune system. To observe whether 5Aza would affect tumor development via adaptive immunity, the Rag2 knockout mouse model with lymphocyte deficiency was employed. We found that 5AZA-mediated tumor inhibition disappeared in Rag2 mice (Figure 1I and Figure S2B). The same phenomenon was also observed in anti-CD8 and anti-CD4 neutralizing antibody therapy trials (Figure 1J and Figure S2C), indicating 5Aza inhibition of CRC-PC progression via lymphocytes. Meanwhile, we also found that the inhibitory effect of 5Aza on the CRC-PC disappeared in macrophage^ko^ mice (Figure 1K-L and Figure S2D-E). More importantly, adoptive therapy of peritoneal macrophages pretreated with 5Aza could obviously inhibit the progression of subcutaneous growth or peritoneal metastasis of CRC (Figure 1M-N and Figure S2F-G). These results indicated that the 5Aza-mediated suppression of CRC-PC was dependent on lymphocytes and macrophages.

### 5Aza activates M1-like macrophages in CRC-PC

**Figure 2:**
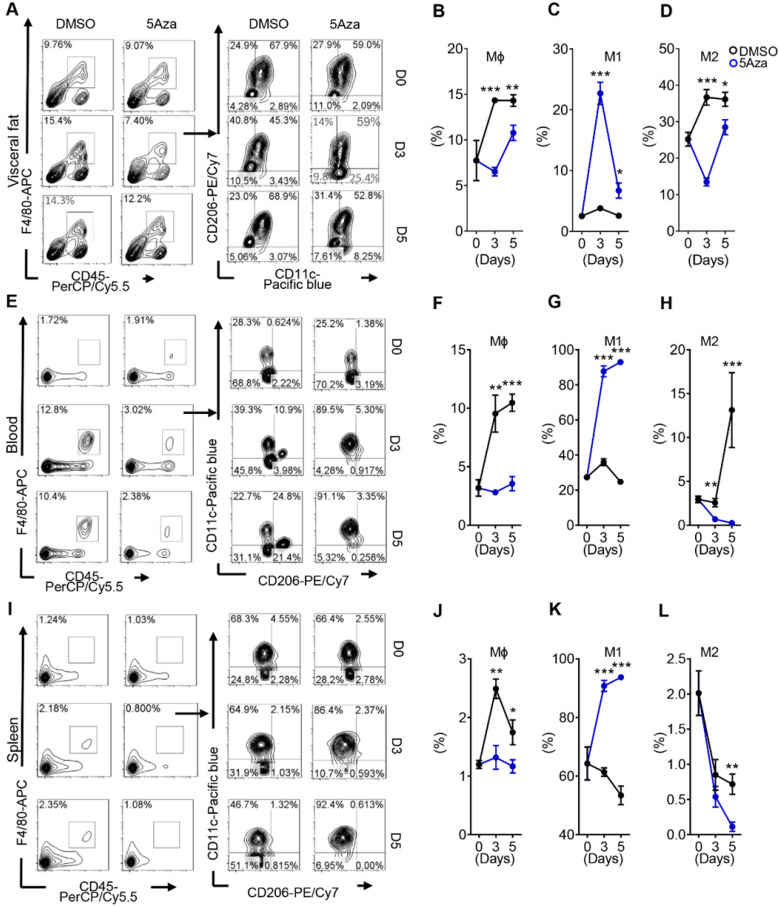
** 5Aza activates M1-like macrophages in CRC-PC. A,** Macrophage infiltration in visceral fat. Six-week-old male mice were intraperitoneally injected with MC38 cells (1.0×10^6^ cells in 100 µL PBS) and immediately treated with DMSO or 5Aza. Then, the stromal vascular fraction (SVF) cells of the epididymal fats were isolated on days 0, 3 and 5. Macrophages (CD45^+^F4/80^+^CD3^-^), M1-like cells (CD45^+^F4/80^+^CD3^-^CD11c^+^CD206^-^), and M2-like cells (CD45^+^F4/80^+^CD3^-^CD11c^-^CD206^+^) were dynamically calculated by flow cytometry. This experiment was repeated three times. Each tested sample was pooled from 3 individual ones. Representative result was shown. **B,** Calculation of macrophages in SVF cells as described above in A (n = 3). **C,** Calculation of M1-like macrophages in SVF cells as described above in A (n = 3). **D,** Calculation of M2-like macrophages in SVF cells as described above in A (n = 3). **E-H,** Frequencies of total (F), M1-like (G) and M2-like (H) macrophages in peripheral blood of the mice as described above in A. Representative result was shown. **I-L,** Frequencies of total (J), M1-like (K) and M2-like (L) macrophages in spleen of the mice as described above in A. Representative result was shown. All the data were shown as the mean ± s.e.m. All data were analyzed with Student's t test (*P < 0.05, **P < 0.01, and ***P < 0.005).

Macrophages account for the largest proportion of myeloid infiltrate in the tumor microenvironment (TME), and are closely associated with tumor growth, invasion, metastasis, and poor prognosis in cancer patients 23. Therefore, we observed whether the activity of macrophages in the TME was influenced by 5Aza in CRC-PC in immune competent mice. As previously reported, CRC cells mainly colonized in visceral fat. Therefore, we first examined the changes of visceral fat macrophages. The results showed that the infiltration of total macrophages was obviously reduced in the visceral fat of 5Aza-treated mice (Figure 2A-B and Figure S3A). In particular, the ratios of M1-like macrophages obviously increased over time after 5Aza treatment, whereas the ratios of M2-like macrophages decreased significantly (Figure 2C-D). Systemic immunity is critical for antitumor response in tumor metastasis 24. Next, we investigated the immune status in peripheral blood and spleens from the mice engrafted with MC38 tumors. The results showed that frequencies of M1-like macrophages were notably increased, while the total and M2-like macrophages declined over time in response to 5Aza treatment in the blood (Figure 2E-H) and spleens (Figure 2I-L). To further validate aforementioned findings, we established PC models using another colorectal cancer cell line CT26. Similar results were observed in the CT26-based PC models (Figure S3B-I). These findings indicated that 5Aza could activate M1-like macrophages in CRC-PC mice.

### 5Aza regulates T cell frequencies in CRC-PC

**Figure 3:**
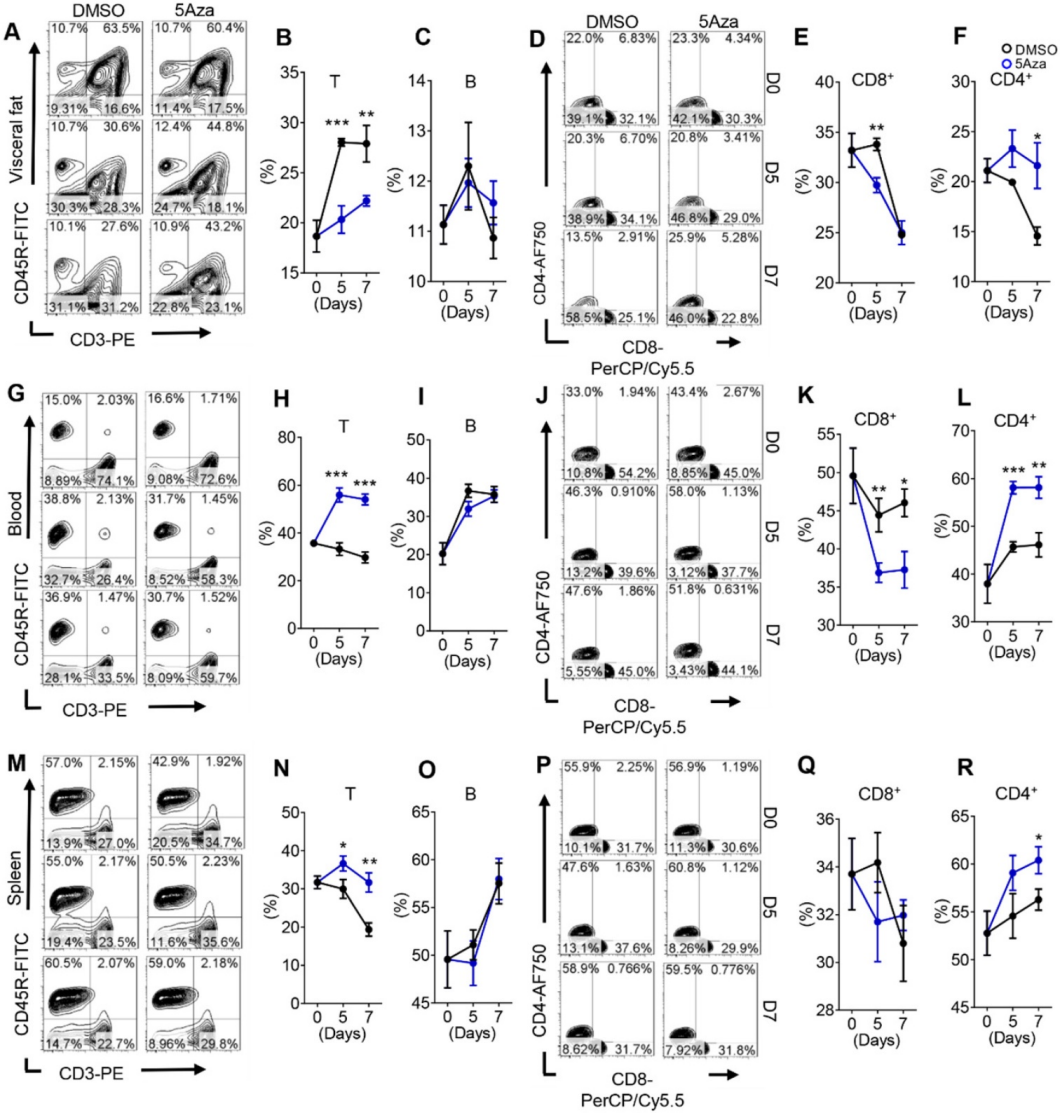
** 5Aza regulates T cell frequencies in CRC-PC. A,** Lymphocyte infiltration in visceral fat. Six-week-old male mice were intraperitoneally injected with MC38 cells (1.0×10^6^ cells in 100 µL PBS) and immediately treated with DMSO or 5Aza. Then, the SVF cells of the epididymal fats were isolated on days 0, 5 and 7. T cells (DAPI^-^CD45^+^CD3^+^CD45R^-^), B cells (DAPI^-^CD45^+^CD3^-^CD45R^+^), CD8^+^T cells (DAPI^-^CD45^+^CD3^+^CD45R^-^CD8^+^CD4^-^), and CD4^+^T cells (DAPI^-^CD45^+^CD3^+^CD45R^-^CD8^-^CD4^+^) were dynamically calculated by flow cytometry. This experiment was repeated three times. Each tested sample was pooled from 3 individual ones. Representative result was shown. **B-C,** Calculation of T cells (B) and B cells (C) in SVF cells as described above in A (n = 3). **D-F,** Calculation of CD8^+^ (E) and CD4^+^ (F) T cells in SVF cells as described above in A (n = 3). **G-L,** Frequencies of total T (H), B (I), CD8^+^ T (K) and CD4^+^T (L) cells in peripheral blood of the mice as described above in A. Representative result was shown. **M-R,** Frequencies of total T (N), B (O), CD8^+^ T (Q) and CD4^+^T (R) cells in spleen of the mice as described above in A. Representative result was shown. All the data were shown as the mean ± s.e.m. All data were analyzed with Student's t test (*P < 0.05, **P < 0.01, and ***P < 0.005).

Adaptive immunity and innate immunity often play a synergistic role in suppressing cancer progression 25. Aforementioned results indicated that lymphocytes were involved in the anti-tumor effect of 5Aza. Thus we dynamically analyzed the lymphocyte changes in tumor-seeded mice. As expected, the results showed that 5Aza regulated lymphocyte infiltration in visceral fat at the early stage of CRC-PC (Figure 3A and Figure S4A). In particular, the proportion of total T cells decreased significantly while B cells remained unchanged in response to 5Aza treatment (Figure 3B-C). Furthermore, the frequencies of CD4^+^ T cells increased significantly over time after 5Aza treatment, whereas the ratios of CD8^+^ T cells had a descending trend (Figure 3D-F).

Note-worthily, the number of T cells in the blood and spleen increased rapidly in response to 5Aza treatment, while the number of B cells was also not altered (Figure 3G-I and 3M-O). In detail, the ratios of CD4^+^ T cells increased notably over time after 5Aza treatment, whereas the ratios of CD8^+^ T cells had an opposite trend (Figure 3J-L and 3P-R). Similar results were further obtained in CT26-engrafted PC models (Figure S4B-I). In addition, 5Aza did not affect Tregs (Figure S4J). Taken together, these results suggest that 5Aza regulates T cell frequencies in CRC-PC.

### 5Aza potentiates macrophage-dependent T-cell activation

**Figure 4:**
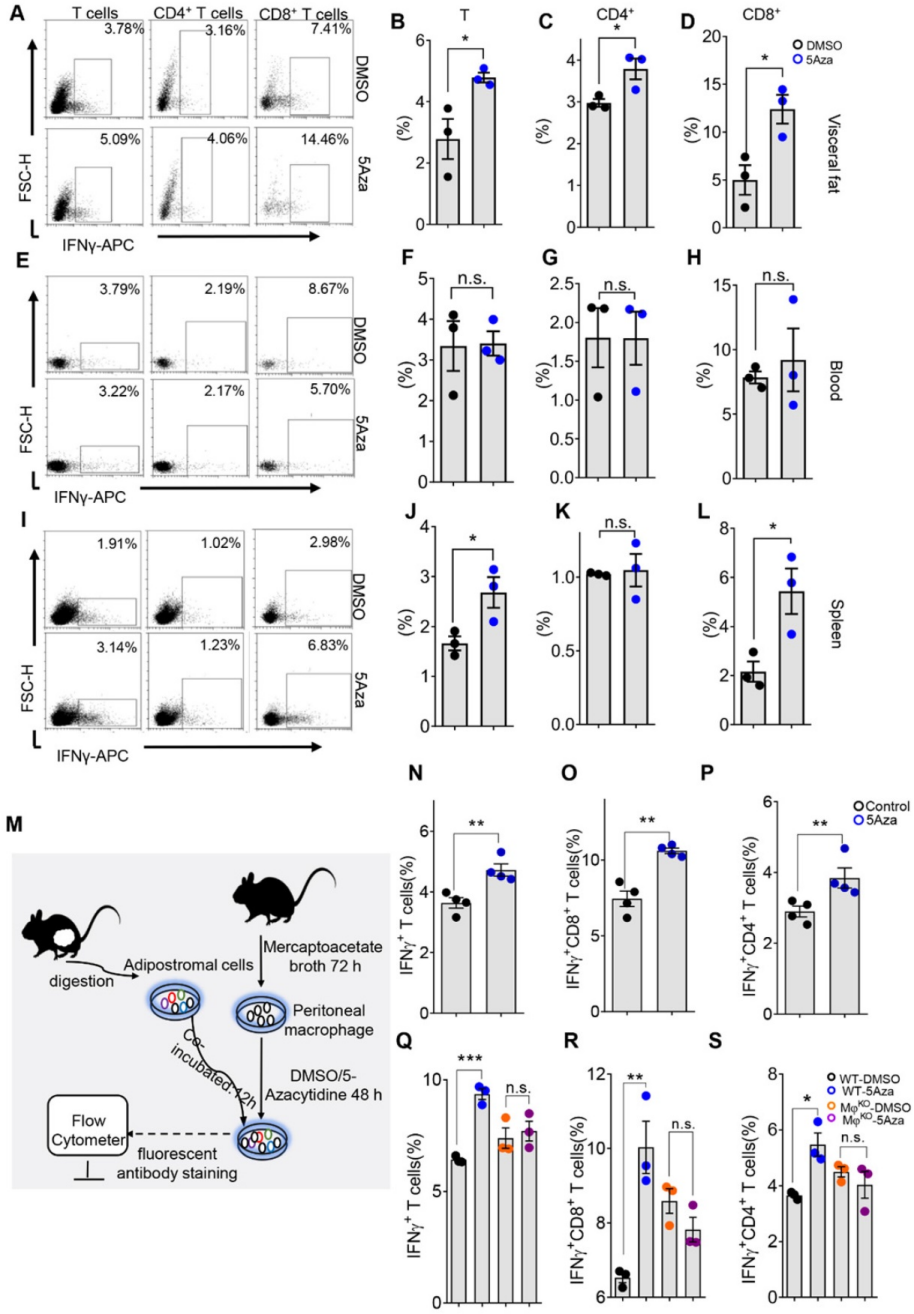
** 5Aza potentiates macrophage-dependent T-cell activation. A,** Percentage of IFNγ^+^ cells in visceral fat. Six-week-old male mice were intraperitoneally injected with MC38 cells (1.0×10^6^ cells in 100 µL PBS) and immediately treated with DMSO or 5Aza. Then, the SVF cells of the epididymal fats were isolated on day 5. After 6-8 h activation by cell activation cocktail, IFNγ^+^ T cells (DAPI^-^CD45^+^CD3^+^ IFNγ^+^), IFNγ^+^ CD8^+^ T cells (DAPI^-^CD45^+^CD3^+^CD8^+^CD4^-^IFNγ^+^), and IFNγ^+^CD4^+^T cells (DAPI^-^CD45^+^CD3^+^CD8^-^CD4^+^ IFNγ^+^) were dynamically calculated by flow cytometry. This experiment was repeated three times. Each tested sample was pooled from 3 individual ones. Representative result was shown. **B-D,** Calculation of IFNγ^+^ T cells (B), IFNγ^+^CD4^+^ T cells (C) and IFNγ^+^CD8^+^ T cells (D) in SVF cells as described above in A (n = 3). **E-H,** Frequencies of IFNγ^+^ T cells (F), IFNγ^+^CD4^+^ T cells (G) and IFNγ^+^CD8^+^ T cells (H) in peripheral blood of the mice as described above in A. Representative result was shown. **I-L,** Frequencies of IFNγ^+^ T cells (J), IFNγ^+^CD4^+^ T cells (K) and IFNγ^+^CD8^+^ T cells (L) in spleen of the mice as described above in A. Representative result was shown. **M,** Schematic diagram of lymphocytes stimulated by macrophages *in vitro*. After 72 h of thioglycolate induction, mice were sacrificed and peritoneal macrophages (PMs) were harvested and treated with DMSO or 5Aza for 48 h. Then, SVFs of the epididymal fats were added to co-culture and the expression of IFNγ^+^ T cells were analyzed. **N-P,** Calculation of IFNγ^+^ T cells (N), IFNγ^+^CD8^+^ T cells (O) and IFNγ^+^CD4^+^ T cells (P) as described above in M (n = 3). **Q-S,** Calculation of IFNγ^+^ T cells. The SVFs of the epididymal fats from macrophage^ko^ were treated with DMSO or 5Aza (4 µM), and then the levels of IFNγ^+^ cells (Q), IFNγ^+^CD8^+^ T cells (R) and IFNγ^+^CD4^+^ T cells (S) were analyzed by flow cytometry (n = 3). Representative result was shown. All the data were shown as the mean ± s.e.m. All data were analyzed with Student's t test. (n.s., not significant; *P < 0.05, **P < 0.01, and ***P < 0.005).

Interferon-γ (IFNγ) plays a key role in activating cellular immunity and thus stimulating anti-tumor immune responses. Based on its pro-apoptotic and anti-proliferative functions, IFNγ is considered to have potential adjuvant immunotherapeutic effects on different types of cancer 26. CD8^+^ and CD4^+^ T cells are the main paracrine sources of IFNγ, which may indicate the anti-tumor activity of T cells to some extent 27. Therefore, we further investigated the secretion level of IFNγ in T cells. We found that the IFNγ levels of total, CD4^+^ and CD8^+^ T cells in visceral fat increased on day 5 (Figure 4A-D), suggesting 5Aza's potential role in enhancing the anti-tumor activity of T cells. Interestingly, the levels of IFN-γ in T, CD4^+^ and CD8^+^ T cells in blood were not changed in response to 5Aza treatment (Figure 4E-H). Although there was also no change in IFN-γ^+^CD4^+^ T cells in spleen, IFN-γ^+^ and IFN-γ^+^CD8^+^ T cells were significantly elevated in response to 5Aza treatment (Figure 4I-L). The comparable results of IFNγ expression in T cells were also validated in the CT26 cell-based PC models (Figure S5A-D).

Macrophages are important regulators of T cell activation. In TME, macrophages, especially M1-like macrophages, mediate and provide costimulatory signals and cytokine secretion required for effective T cell activation 28. To further explore the relationship between 5Aza-mediated macrophage polarization and T cell activation, we designed an *ex vivo* experiment as shown in Figure 4M. As expected, compared with DMSO-pretreated peritoneal macrophages (PMs), 5Aza-pretreated PMs could promote the secretion of IFNγ from visceral adipose T cells as well as the CD4^+^ and CD8^+^ subpopulations (Figure 4N-P and Figure S5E). Additionally, we demonstrated that 5Aza-potentiated expression of IFNγ in visceral adipose T cells, especially in the CD8^+^ T cells, was fully prevented in macrophage^ko^ mice (Figure 4Q-S and Figure S5F). Those results indicated that 5Aza stimulated macrophage-dependent T-cell activation in CRC-PC in mice.

### 5Aza stimulates p65-dependent macrophage activation and tumor suppression

**Figure 5:**
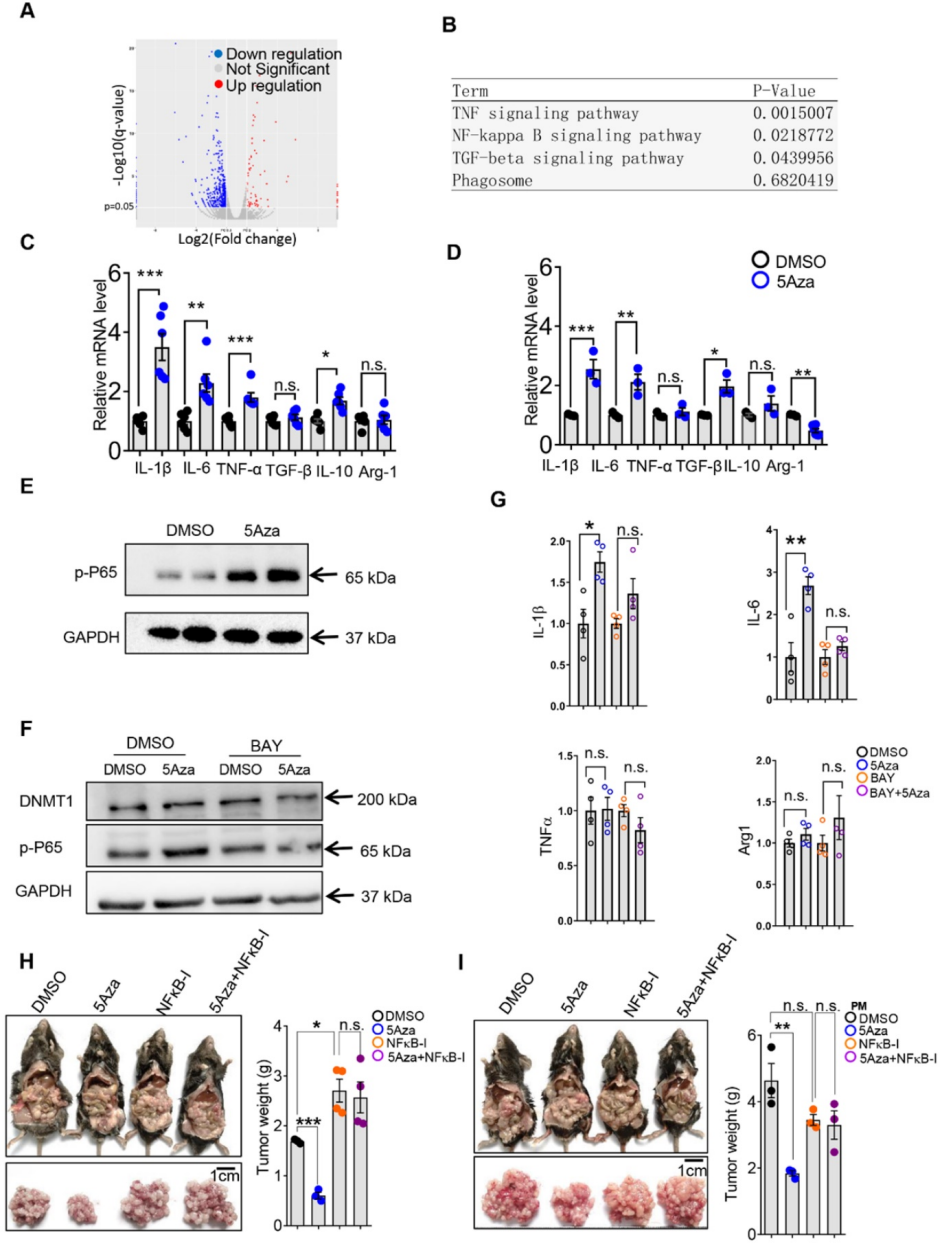
** 5Aza stimulates p65 phosphorylation-dependent macrophage activation and tumor suppression. A,** Fold changes of gene expression in primary mouse macrophages treated with 5Aza or DMSO according to mRNA sequencing. Red points indicated upregulated genes; Blue points indicated downregulated genes; Grey points showed the unchanged genes. **B,** Enrichment of signal pathways between DMSO and 5Aza group as described in A. **C-D,** mRNA levels of cytokines in PMs (C) and bone marrow-derived macrophages (BMDMs) (D) treated with DMSO or 5Aza (4 µM) for 48 h. **E,** Immunoblotting assays of phosphorylated p65 (p-p65) in PMs treated with DMSO or 5Aza (4 µM) for 48 h. **F,** PMs were treated with DMSO or 5Aza (4 µM) for 36 h and then additionally co-stimulated by the NF-κB inhibitor BAY 11-7082 (10 µM) for 12 h before being harvested for immunoblotting assays. **G,** mRNA levels of cytokines in PMs treated with DMSO or 5Aza (4 µM) for 36 h, and then plus with BAY 11-7082 (10 µM) for 12 h. **H,** Six-week-old male WT mice were intraperitoneally injected with MC38 cells (1.0×10^6^ cells in 100 µL PBS) and immediately treated with DMSO or 5Aza on days 0, 2, and 4 plus with BAY (20 mg/kg). Tumor nodules were observed on day 14. Representative result was shown (n = 3). **I,** 2.0×10^5^ PMs was pretreated with DMSO or 5Aza for 36 h plus with BAY 11-7082 (10 µM) for 12 h, and then injected into the abdominal cavity of mice together with MC38 cells (1.0×10^6^ cells in 100 µL PBS for each mouse). Tumor nodules were observed on day 14. Representative result was shown (n = 3). All the data were shown as the mean ± s.e.m. All data were analyzed with Student's t test (n.s., not significant; *P < 0.05, **P < 0.01, and ***P < 0.005).

The aforementioned results showed that 5Aza could promote macrophage polarization and subsequent activation of T cells. Then, we investigated how 5Aza stimulated macrophage activation. According to RNA sequencing and signals enrichment analyses, inflammation-related signaling pathways of PMs in response to 5Aza treatment were highly enriched (Figure 5A-B). We further validated that the 5Aza-pretreated PMs expressed more proinflammatory cytokines, such as IL-1β, and IL-6, than the DMSO-pretreated PMs (Figure 5C). Similar results were obtained in bone marrow-derived macrophages (BMDMs) (Figure 5D). As shown in Figure 5B, NF-κB signaling might be downstream of 5Aza. As expected, we found that 5Aza obviously stimulated the phosphorylation of p65 in PMs (Figure 5E) and BMDMs (Figure S6A). The levels of p65 phosphorylation in PMs increased dramatically and were reversed by an NF-κB inhibitor (Figure 5F). Subsequently, 5Aza-mediated upregulation of pro-inflammatory cytokines, such as IL-1β and IL-6, were prevented by the NF-κB inhibitor to some extent (Figure 5G). These results indicated that 5Aza promoted p65-dependent M1-like polarization of macrophages. Functionally, we demonstrated that the 5Aza-suppressed CRC-PC was fully abolished by the treatment of NF-κB inhibitor (Figure 5H). Similar results were obtained when we performed adoptive therapy in CRC-PC mice by using 5Aza and the NF-κB inhibitor-pretreated PMs (Figure 5I).

In addition, macrophage phagocytosis also plays an important role in anti-tumor response 29. However, we found that 5Aza did not affect macrophage phagocytosis (Figure S6B). Our previous study revealed that NF-κB signal-dependent MMP production could regulate cancer metastasis 30. However, 5Aza did not alter the expression of MMPs in RNA sequencing assays (Figure S6C), although 5Aza activated p65 NF-κB signal (Figure 5E and Figure S6A).

As a specific inhibitor of DNMT1, the function of 5Aza may be mainly dependent on DNMT1 31. Therefore, we further investigated whether the M1 polarization induced by 5Aza was mediated by DNMT1. However, we found that 5Aza did not regulate DNMT1 expression (Figure 5F). Instead of promoting p65 phosphorylation, DNMT1 silence in macrophages obviously decreased it (Figure S7A-B). Similarly, DNMT1 knockdown inhibited the pro-inflammatory cytokines, such as IL6 (Figure S7C). These results indicated that 5Aza-stimulated M1 activation is independent of DNMT1.

### 5Aza targeting ABC A9 promotes cholesterol accumulation, macrophage polarization and CRC-PC suppression

**Figure 6:**
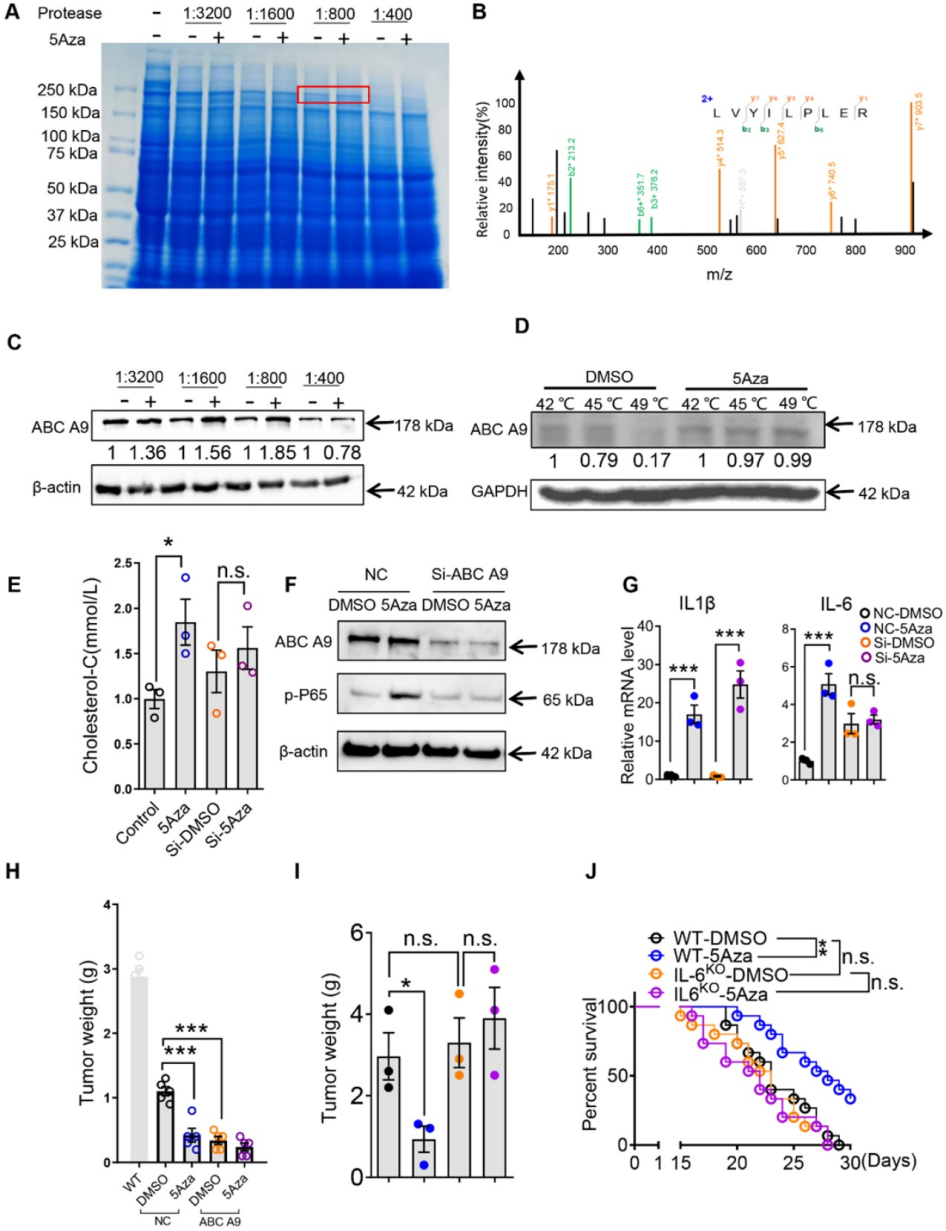
** 5Aza targeting ABC A9 promotes cholesterol accumulation, macrophage polarization and CRC-PC suppression. A,** Coomassie blue of DARTS assay. The band with molecular weight around 200 kDa protected by 5Aza (400 µM) was indicated by red frame. **B,** Adapted image of mass spectra for ABC A9. **C,** DARTS and Western blot to confirm 5Aza combined with ABC A9. **D,** CETSA and Western blot to confirm 5Aza combined with ABC A9. **E,** Cholesterol content in BMDMs. The BMDMs pre-infected with an ABC A9 silencing plasmid and then treated with DMSO or 5Aza (4 µM) for 48 h. **F,** Immunoblotting assays of p-p65 in BMDMs. The BMDMs pre-infected with an ABC A9 silencing plasmid and then treated with DMSO or 5Aza (4 µM) for 48 h. **G,** mRNA levels of cytokines in BMDMs as described in D. **H,** Weight of the tumor nodes. Six-week-old male WT were intraperitoneally inoculated with MC38 cells (1.0×10^6^ cells in 100 µL PBS per mouse) on day 0 and then administered with Si-NC/ABC A9 BMDMs. Meanwhile, these mice were treated with 5Aza on days 0, 2, and 4. Tumor nodules were observed on day 14. Representative result was shown (n = 3). **I,** Six-week-old male WT or IL-6^KO^ mice were intraperitoneally inoculated with MC38 cells (1.0×10^6^ cells in 100 µL PBS per mouse) on day 0 and then administered with 5Aza on days 0, 2, and 4. Tumor nodules were observed on day 14. Representative result was shown (n = 3). **J,** The survival of tumor-seeded mice treated with 5Aza on days 0, 2, and 4. Tumor-seeded models were established as described in F. This experiment was repeated three times (n =15).

These findings lead us to propose that 5Aza may have an unknown target that mediates its role in promoting p65 phosphorylation through previously unknown mechanisms. To address this question, we performed Drug Affinities Target Stability (DARTS) test to explore the unrecognized mechanism. Firstly, the PM lysates were mixed with different concentrations of 5Aza at 37 °C for 30 min, and proteases at different concentrations were added at room temperature for 10 min. Sodium dodecyl sulfate polyacrylamide gel electrophoresis (SDS-PAGE) was used to isolate the proteins and staining with coomassie bright blue. The results showed that 5Aza protected a band with a molecular weight of about 200 kDa (Figure 6A). The band was excised for protein identification by mass spectrometry, and the results showed that ABC A9 was a potential target of 5Aza with the highest score (Figure 6B). We further conducted western blot with DARTS samples and found that 5Aza could protect ABC A9 (Figure 6C). In order to further confirm the associations of 5Aza with ABC A9, we employed the cellular thermal shift assay (CETSA) which are used to study thermal stabilization of proteins upon ligand binding 22. CETSA results clearly indicated that 5Aza can bind with ABC A9 (Figure 6D). ABC A9 belongs to ABC A transporter subfamily and is mainly distributed on membrane structures. ABC A9 was found to be involved in monocyte differentiation and macrophage lipid homeostasis, especially cholesterol 15. Cholesterol accumulation is associated with M1-like macrophages 32, 33, while cholesterol efflux potentiates M2 activation 16. Therefore, we further tested the cholesterol content of macrophages treated with 5Aza. We found that the effect of 5Aza-mediated increase of cholesterol content disappeared in BMDMs transfected with si-ABC A9 (Figure 6E), suggesting that the binding of 5Aza and ABC A9 causes the accumulation of cholesterol in macrophages. Meanwhile, we found that 5Aza didn't change the protein level of ABC A9 (Figure 6F), indicating that 5Aza may inhibit ABC A9's activity in cholesterol efflux. Note-worthily, 5Aza-mediated p65 phosphorylation was absent in si-ABCA9-transfected BMDMs (Figure 6F). The same phenomenon occurred in the 5Aza-mediated elevation of IL-6, although IL-1β was still elevated (Figure 6G). Importantly, we found that BMDMs with ABC A9 knockdown can be adopted to treat CRC-PC (Figure 6H and Figure S8A). Furthermore, we found that 5Aza-mediated tumor inhibition was abrogated in IL-6^KO^ mice (Figure 6I-J and Figure S8B). Taken together, those results indicate that 5Aza binds with ABC A9 to promote cholesterol accumulation, p65 phosphorylation and IL-6 expression in macrophages, which finally inhibits CRC-PC.

### 5Aza synergizes chemotherapy of CRC-PC in mice

**Figure 7:**
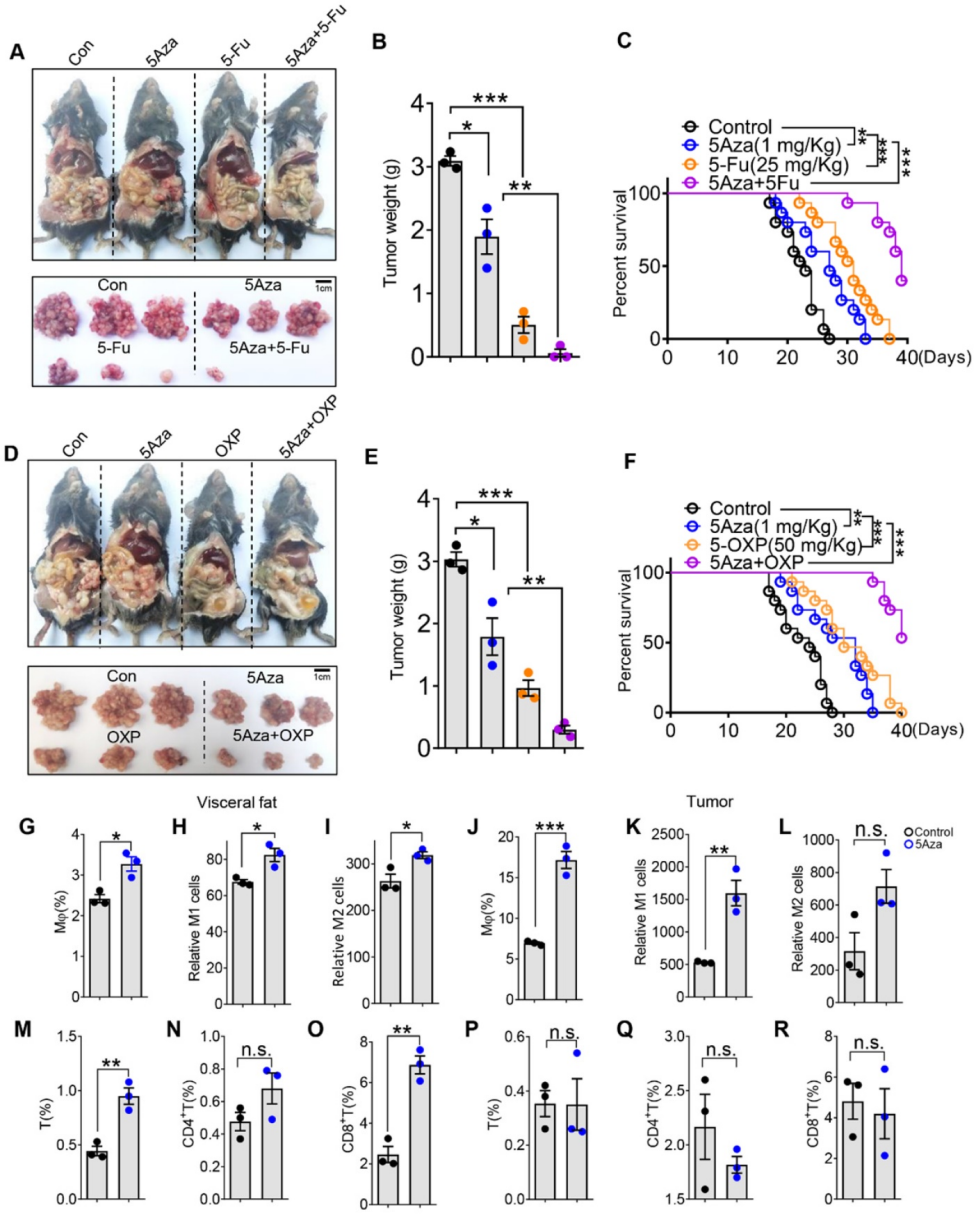
**5Aza synergizes chemotherapy of CRC-PC in mice and regulates activity of stromal macrophages and T cells in CRC patients. A-B,** Six-week-old male WT mice were intraperitoneally inoculated with MC38 cells (1.0×10^6^ cells in 100 µL PBS per mouse) on day 0 and then administered with 5Aza and/or 5-fluorouracil (5Fu, 50 mg/kg) on days 0, 2, and 4. Tumor nodules were observed on day 14. Representative result was shown (n = 3). **C,** The survival of tumor-seeded mice treated with 5Aza and/or 5Fu (50 mg/kg) on days 0, 2, and 4. Tumor-seeded models were established as described in A. This experiment was repeated three times (n = 15). **D-E,** Six-week-old male WT mice were intraperitoneally injected with MC38 cells (1.0×10^6^ cells in 100 µL of PBS per mouse) on day 0 and then administered with 5Aza and/or oxaliplatin (OXP, 5 mg/kg) on days 0, 2, and 4. Tumor nodules were observed on day 14. Representative result was shown (n = 3). **F,** The survival of tumor-seeded mice treated with 5Aza and/or OXP (5 mg/kg) on days 0, 2, and 4. Tumor-seeded models were established as described in D. This experiment was repeated three times (n =15). **G-I,** Calculation of total (G), M1-like (H) and M2-like (I) macrophages in omental fats. Stromal cells of human visceral fats were collected and stimulated by DMSO or 5Aza for 48 h. Then macrophages and the subpopulations were analyzed by flow cytometry (n = 3). **J-L,** Calculation of total (J), M1-like (K) and M2-like (L) macrophages in human CRC tissues. Stromal cells of human CRC tissues were collected and stimulated by DMSO or 5Aza for 48 h. Then macrophages and the subpopulations were analyzed by flow cytometry (n = 3). **M-O,** Calculation of IFNγ^+^ T cells (M), FNγ^+^ CD4^+^ T cells (N) and FNγ^+^ CD8^+^ T cells (O) in the stromal cells as described above in G (n = 3). **P-R,** Calculation of IFNγ^+^ T cells (P), IFNγ^+^CD4^+^ T cells (Q) and IFNγ^+^CD8^+^ T cells (R) in human CRC tissues as described above in J. All the data were shown as the mean ± s.e.m. The data in B, E and G-R were analyzed with Student's t test. The data in C and F were analyzed with Gehan-Breslow-Wilcoxon test (n.s., not significant; *P < 0.05, **P < 0.01, and ***P < 0.005).

At present, the main treatment for CRC-PC relies on surgery, radiotherapy and chemotherapy. 5-fluorouracil (5Fu) and oxaliplatin (OXP) are conventional chemotherapy drugs. We further investigated whether 5Aza could synergizes chemotherapy to treat CRC-PC. As expected, we found that 5Fu markedly inhibited CRC-PC, and this effect was further enhanced by the addition of 5Aza (Figure 7A-B). Moreover, 5Aza plus 5Fu synergistically improved the survival of tumor-seeded mice (Figure 7C). Similarly, we also found a strong synergistic effect between 5Aza and OXP, namely inhibiting CRC-PC and improving survival (Figure 7D-F).

### 5Aza activates macrophages and T cells in omental fats from CRC patients

To further investigate the relevance of our findings to human pathophysiology, we next explored whether 5Aza could regulate macrophage polarization and T cells activity from CRC patients. We demonstrated that 5Aza treatment obviously increased the frequencies of total, M1-like and M2-like macrophages in the stromal vascular fraction of omental fats from CRC patients (Figure 7G-I and Figure S9A-B). Similar results were obtained in the stromal cells of CRC tissues in response to 5Aza treatment (Figure 7J-L and Figure S9A-B). We further revealed that 5Aza treatment stimulated the secretion of IFNγ in total, CD4^+^ and CD8^+^ T cells in the stromal cells of omental fats from CRC patients (Figure 7M-O and Figure S9C-D). However, 5Aza did not activate total, CD4^+^ or CD8^+^ T cells in the stromal cells of patient CRC tissues (Figure 7P-7R and Figure S9C-D). These results indicated that compared with tumor tissues, anti-tumor immunity in adipose tissues was more easily activated by 5Aza. This also suggested that 5Aza might not be effective on the primary CRC, but might have a better clearance effect on the metastases in the visceral fats.

## Discussion

CRC-PC is a terminal phase with limited therapeutic strategies 34. In the present study, we found that 5Aza targeting ABC A9 promotes macrophage cholesterol accumulation, which in turn increases p65-dependent IL-6 expression, and then activates T cells to inhibit CRC-PC. Importantly, we also found that 5Aza could cooperate with conventional chemotherapy drugs to inhibit the progression of CRC-PC and prolong the survival of tumor-seeded mice. The anti-tumor immunity induced by 5Aza was further verified in *ex vivo* experiments by using CRC patient-derived fat and tumor tissues.

Previous studies have revealed that 5Aza can directly mediate the apoptosis of various types of tumor cells and inhibit tumor progression 35. Although we also found that 5Aza could inhibit the proliferation activity of MC38 cells *in vitro*, it was not observed *in vivo*. Therefore, we speculated that the anti-tumor effect of 5Aza might be mediated by tumor microenvironment. We found that 5Aza could markedly stimulate M1-like macrophages depending on p65. Note-worthily, 5Aza-stimualted p65 activation was independent of DNMT1, although 5Aza was identified as an inhibitor of DNMT1.

We speculate that 5Aza might have some other undefined targets, which might be closely associated with p65 activation. DARTS is a relatively fast and direct way to explore potential protein targets of small molecules. It relies on the principle of protecting the target protein from hydrolysis by proteases by interacting with small molecules 21, 36. Implementation of DARTS assay, we found that ABC A9 may be a potential target of 5Aza. Of course, DARTS can only indicate the protective effect of 5Aza on ABC A9 in the presence of protease, which does not mean that ABC A9 can be up-regulated in live cells. Since ABC A9 belongs to the cholesterol-responsive subclass ABC transporter 15, we further tested the effect of 5Aza on the cholesterol content of macrophages. Interestingly, we found that 5Aza can regulate the cholesterol content of macrophages through ABC A9, which may be related to the obstruction of cholesterol efflux. 5Aza didn't change the protein level of ABC A9, suggesting that the binding of 5Aza and ABC A9 may only impair the cholesterol effluent activity of ABC A9. Cholesterol accumulation is closely related to macrophage inflammation 37, 38, which may explain how ABC A9 regulates p65 phosphorylation. However, 5Aza-mediated IL-1β elevation was not caused by ABC A9, suggesting that 5Aza may have other potential targets. Further exploring the regulatory targets of 5Aza might expand its clinical application.

By using clinical samples, we demonstrated that 5Aza significantly promoted the M1 polarization of fat and CRC-derived macrophages. Especially, 5Aza treatment could largely induce T cell activity in visceral fats, but not in the CRC tissues. We speculated that the tumor tissue-derived T cells might have been exhausted and couldn't be easily reactivated by 5Aza-macrophage axis. However, this finding might provide us a theoretical basis for the clinical treatment of 5Aza in CRC-PC. HIPEC is currently the main treatment for CRC-PC 39. We found that 5Aza could cooperate with OXP and 5Fu to inhibit CRC-PC in mice. We advocate the clinical application of 5Aza in combination with HIPEC.

In summary, our study for the first time reveals that 5Aza binding to ABC A9 promotes cholesterol accumulation and p65-dependent macrophage polarization, which in turn activates T cells and ultimately inhibits CRC-PC. Our findings demonstrate that 5Aza may be a new promising therapeutic drug for CRC-PC and uncover a non-canonical function of 5Aza in regulating cell biology.

## Abbreviations

ABC A9: ATP-binding cassette transporter A9
BMDMs: bone marrow-derived macrophages
CRC: colorectal cancer
DARTS: Drug Affinities Target Stability
DNMT: DNA methyltransferase
HIPEC: hyperthermic intraperitoneal chemotherapy
IFNγ: interferon-γ
OXP: oxaliplatin
PC: peritoneal carcinomatosis
PMs: peritoneal macrophages
5Aza: 5-aza-2'-deoxycytidine
5Fu: 5-fluorouracil
