# Supplementary material for: 5-aza-2′-deoxycytidine potentiates anti-tumor immunity in colorectal peritoneal metastasis by modulating ABC A9-mediated cholesterol accumulation in macrophages
Source: Theranostics. 2022 Jan 1;12(2):875–90. doi: 10.7150/thno.66420 (PMC8692916; doi:10.7150/thno.66420)
Supplement: Supplementary file 1 — Supplementary figures. [file thnov12p0875s1.pdf]

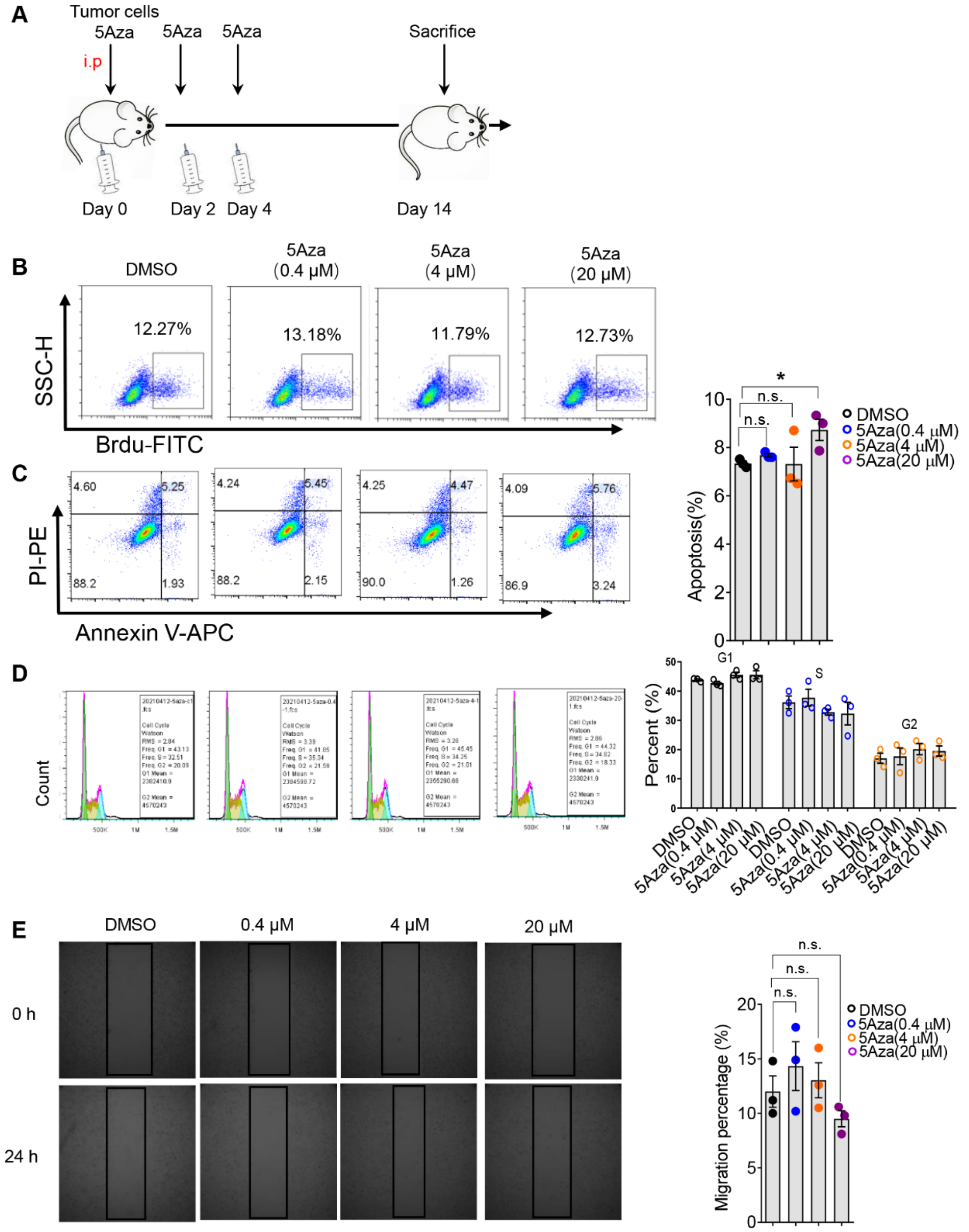

Figure S1. 5Aza does not affect the growth, apoptosis, cycle or migration of MC-38 cells in vitro

A, Schematic of the colorectal peritoneal carcinomatosis model. Six-week-old male mice were intraperitoneally injected with MC38 cells (1.0×10^6/100 μL of PBS) on day 0, and immediately treated with DMSO or 5Aza. Mice were sacrificed for further observation on day 14.

B, Brdu proliferation assay of MC38 treated with different concentrations of 5Aza was analyzed by flow cytometry. (n = 3)

C. Apoptosis of MC38 cells treated with different concentrations of 5Aza was analyzed by flow cytometry. (n = 3)

D, Cell cycle of MC38 cells treated with different concentrations of 5Aza was analyzed by flow cytometry. (n = 3)

E, Wound-healing assays of MC38 cells treated with different concentrations of 5Aza. (n = 3)

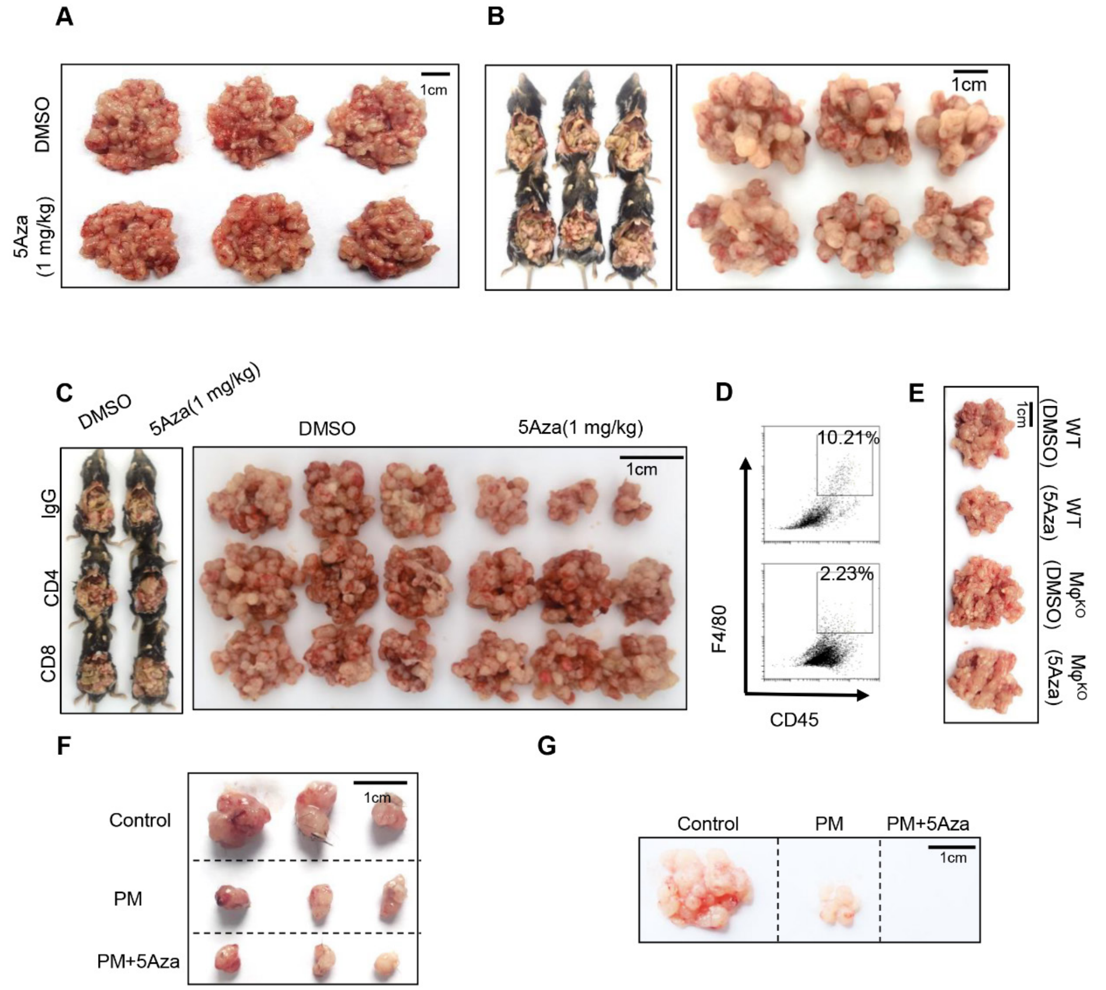

Figure S2. 5Aza suppresses the progression of CRC-PC depending on macrophages and lymphocytes

A, Tumor nodes of MC38 cells from the six-week-old male nude mice treated with DMSO or 5Aza. Tumor-seeded models were established as described in **Figure S1A** on day 0 and the tumor nodules were observed on day 14. Representative result was shown. (n = 3)

B, Tumor nodes of MC38 cells from the six-week-old male Rag2 mice treated with DMSO or 5Aza. Tumor-seeded models were established as described in **Figure S1A** on day 0 and the tumor nodules were observed on day 14. Representative result was shown. (n = 3)

C. Tumor nodes of MC38 cells. Tumor-seeded models were established as described in **Figure S1A** on day 0 and immediately treated with IgG, anti-CD4, or anti-CD8

antibody on days 0, 2, and 4. Tumor nodules were observed on day 14. Representative result was shown. (n = 3)

D. Percentage of visceral fat macrophages.

E, Tumor nodes of MC38 cells from the six-week-old male macrophage^{ko} mice treated with DMSO or 5Aza. Tumor-seeded models were established as described in **Figure S1A** on day 0 and the tumor nodules were observed on day 14. Representative result was shown. (n = 3)

F-G, Six-week-old male mice were subcutaneously (**G**) or intraperitoneally (**H**) engrafted with MC38 cells (5.0 or 1.0×10^6 cells in 100 μL PBS per mouse) on day 0, and together with 1.0 or 0.2×10^5 peritoneal macrophage after treated with DMSO or 5Aza for 48 h. Mice were sacrificed on day 14 and tumor nodules were observed. Representative result was shown. (n = 3)

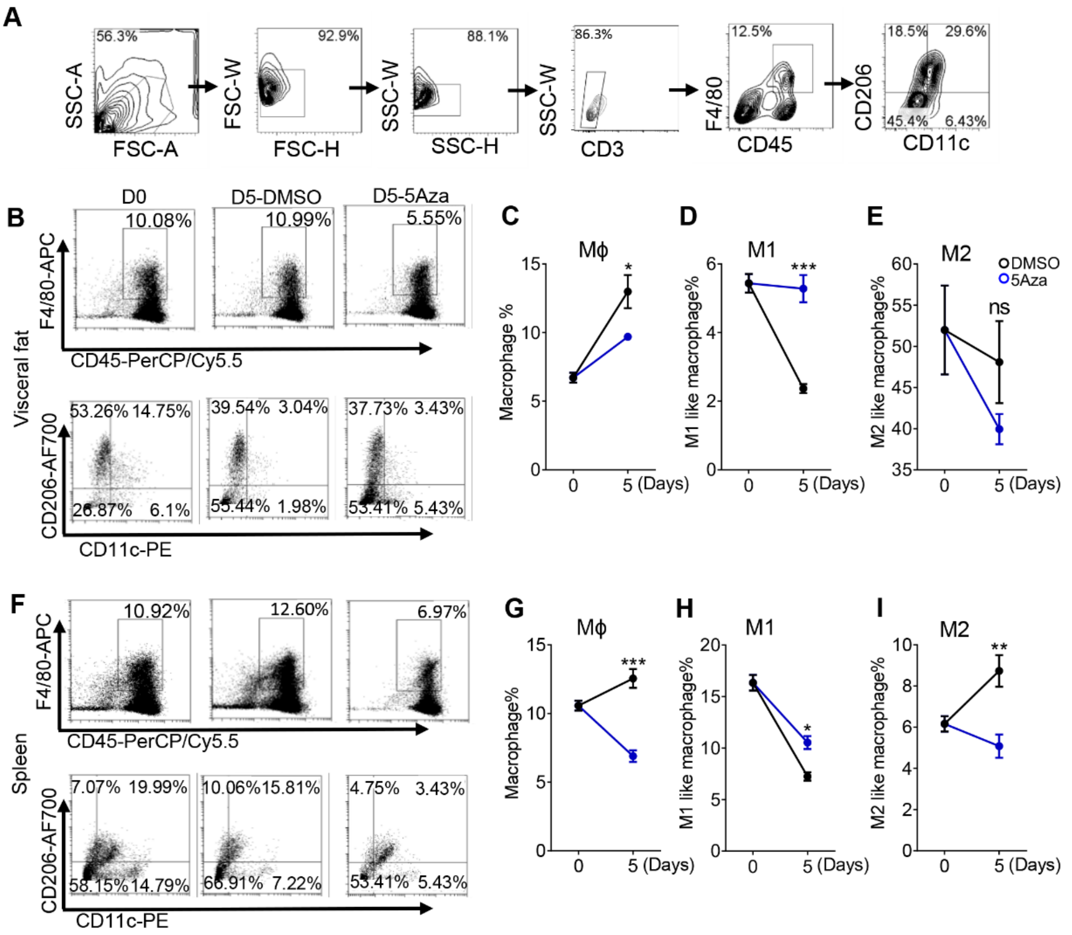

Figure S3. 5Aza stimulates macrophage activity throughout the body in the process of CRC-PC

A, FACS gating strategy for total, M1-like and M2-like macrophages from mouse tissues. Debris and doublets were removed, and macrophages ($CD45^+F4/80^+CD3^-$), M1-like cells ($CD45^+F4/80^+CD3^-CD11c^+CD206^-$), and M2-like cells ($CD45^+F4/80^+CD3^-CD11c^-CD206^+$) were dynamically calculated by flow cytometry.

B-E, Macrophage infiltration in visceral fats. Six-week-old male mice were intraperitoneally injected with CT26 cells (1.0×10^6 cells in 100 μL PBS) and immediately treated with DMSO or 5Aza. Then, the stromal vascular fraction (SVF) cells of the epididymal fats were isolated on days 0 and 5. Macrophages (**C**), M1-like cells (**D**) and M2-like cells (**E**) were dynamically calculated by flow cytometry. This experiment was repeated three times. Each tested sample was pooled from 3 individual ones. Representative result was shown (**B**). (n = 3)

F-I, Frequencies of total (**G**), M1-like (**H**) and M2-like (**I**) macrophages in spleen of

the mice as described above in **A**. Representative result was shown (**F**). (n = 3)
All the data were shown as the mean ± s.e.m and analyzed with Student's t test. (n.s., not significant, *P < 0.05, **P < 0.01, and ***P < 0.005)

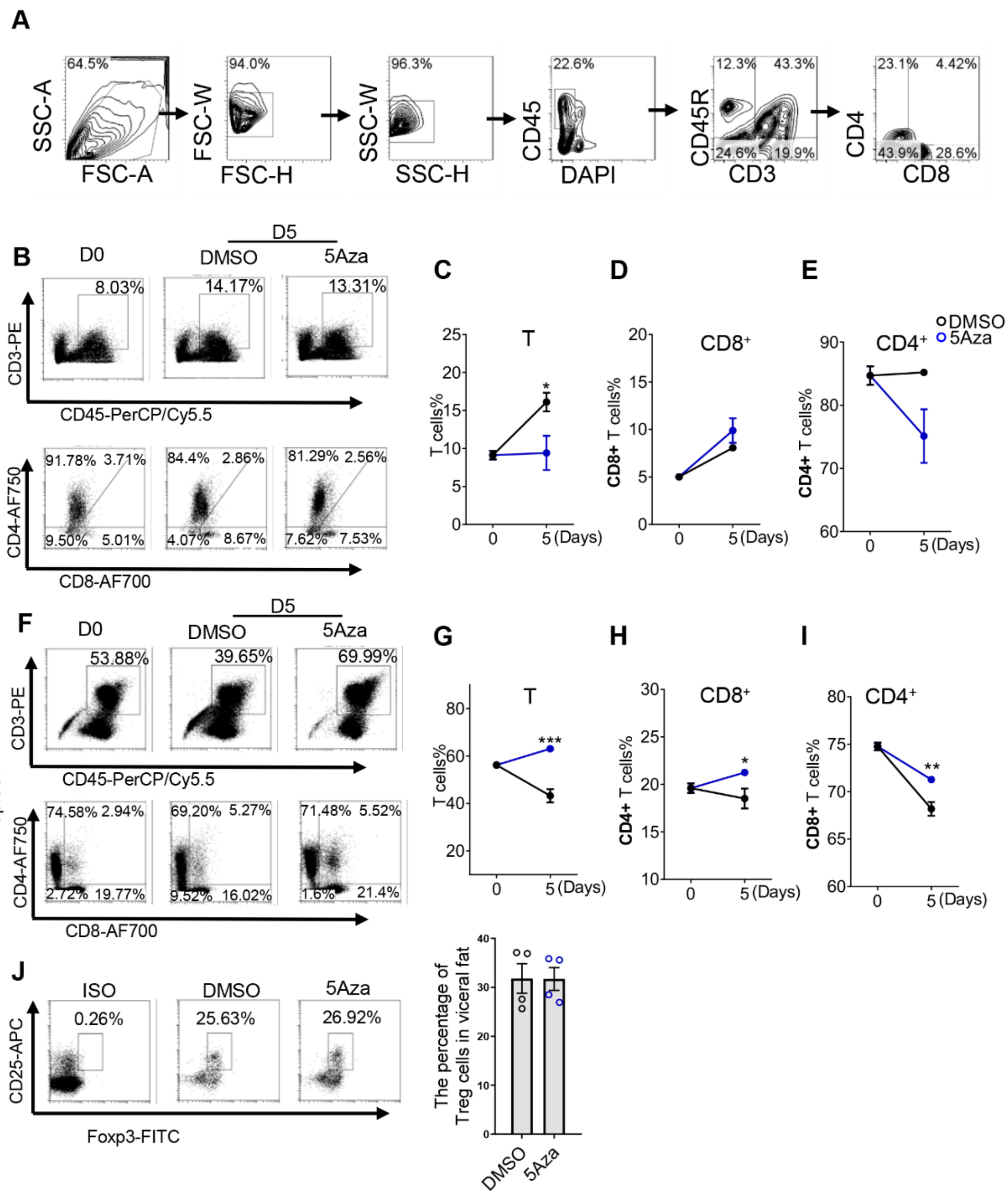

Figure S4. 5Aza regulates systemic T lymphocytes in CRC-PC

A, FACS gating strategy for T cells, CD4$^+$ T cells and CD8$^+$ T cells in mouse tissues. Debris and doublets were removed, and T cells were then assessed as (DAPI$^-$CD45$^+$CD3$^+$), CD8$^+$T cells (DAPI$^-$CD45$^+$CD3$^+$CD8$^+$CD4$^-$) and CD4$^+$T cells (DAPI$^-$CD45$^+$CD3$^+$CD8$^-$CD4$^+$).

B-E, The levels of visceral fat T cells (**C**), CD8$^+$ T cells (**D**) and CD4$^+$ T cells (**E**) from the DMSO or 5Aza-treated mice described in supplementary Figure S2. Representative results were shown. (n = 3)

F-I, The levels of spleen T (**G**), CD8$^+$ T (**H**) and CD4$^+$ T (**I**) cells from the DMSO or 5Aza-treated mice described in supplementary Figure S2. Representative results were shown. (n = 3)

J. The levels of visceral fat Tregs from the DMSO or 5Aza-treated mice described in supplementary Figure S2. Representative results were shown. (n = 3)

All the data were shown as the mean ± s.e.m and were analyzed with Student's t test. (*P < 0.05, **P < 0.01, and ***P < 0.005)

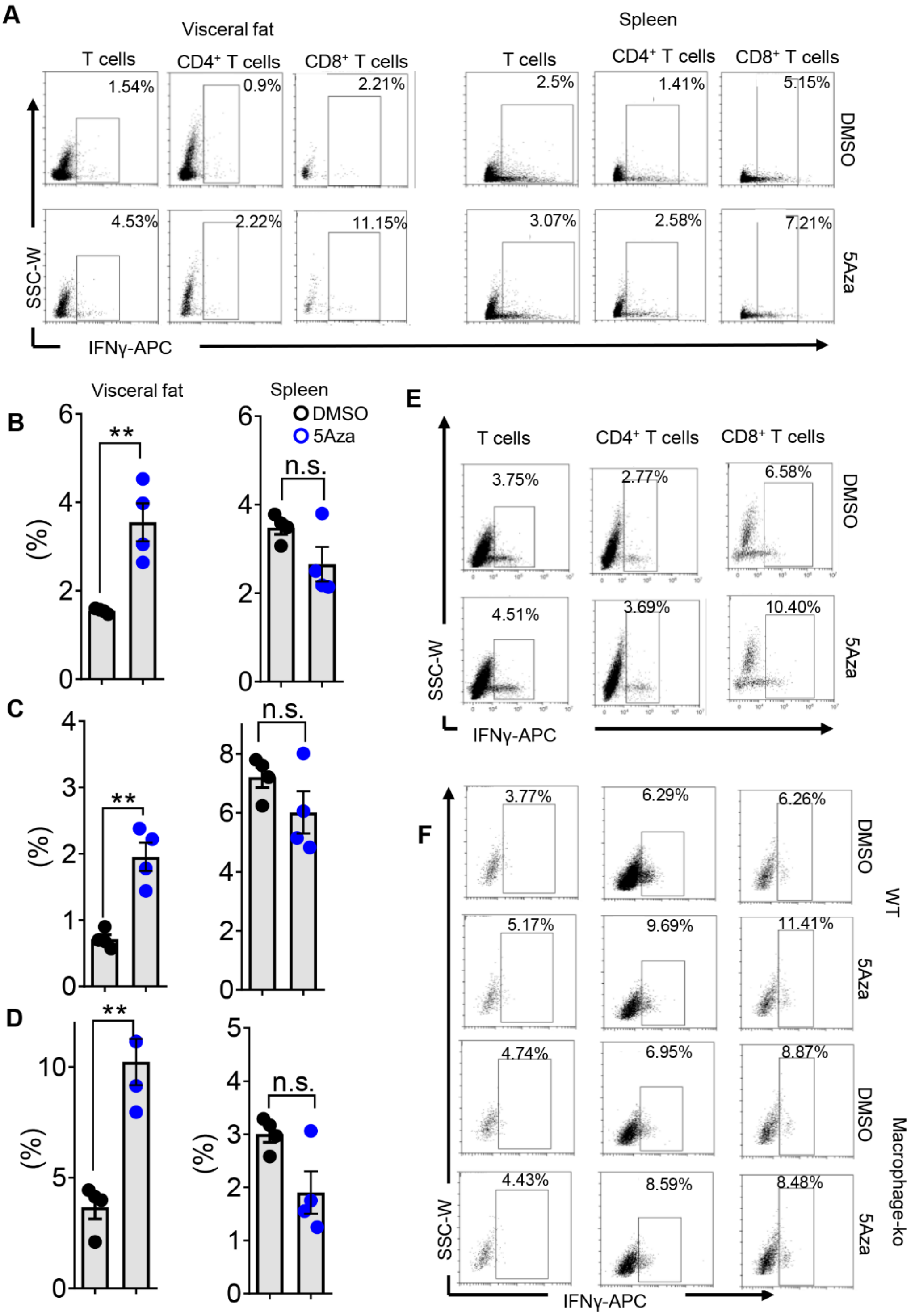

Figure S5. 5Aza potentiates macrophage-dependent T cell activation.

A, The frequencies of visceral fat and spleen IFNγ^{+} T cells, IFNγ^{+}CD4^{+} T cells and IFNγ^{+}CD8^{+} T cells from the tumor-seeded mice treated with DMSO or 5Aza. Six-week-old male Balb/c mice were intraperitoneally inoculated with CT26 cells (1.0

× 10^6 cells in 100 μL of PBS per mouse) on day 0 and then treated with DMSO or 5Aza on days 0, 2, and 4. Then, the stromal cells of the visceral fat and spleen were collected for the analysis of IFNγ$^+$ T cells, CD8$^+$IFNγ$^+$ T cells and CD4$^+$IFNγ$^+$ T cells on day 0 and 5. Representative results were shown.

B, Calculation of IFNγ$^+$ T cells as described above in **A**. (n = 3)

C, Calculation of IFNγ$^+$CD8+ T cells as described above in **A**. (n = 3)

D, Calculation of IFNγ$^+$CD4+ T cells as described above in **A**. (n = 3)

E, The percentage of IFNγ$^+$ T cells. PMs were pretreated with DMSO or 5Aza for 48 h and then additionally co-incubation with the SVFs of the epididymal fats for 6 h. Finally, IFNγ$^+$ T cells, IFNγ$^+$CD8$^+$ T cells and IFNγ$^+$CD4$^+$ T cells from SVFs were analyzed by flow cytometry. Representative results were shown.

F, The ratios of IFNγ$^+$ T cells from visceral fats of macrophage^{ko} mice treated with DMSO or 5Aza for 48 h were analyzed by flow cytometry. Representative results were shown.

All data were analyzed with Student's t test. (n.s., not significant; *P < 0.05, **P < 0.01, and ***P < 0.005)

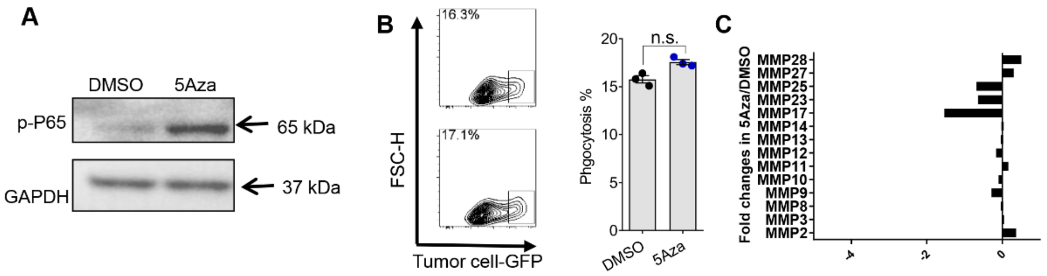

Figure S6. 5Aza does not affect matrix metalloproteinase (MMP) expression or tumor phagocytosis by macrophages

A, Immunoblotting assays of p-p65 in BMDMs treated with 5Aza or DMSO for 48 h.

B, PMs treated with DMSO or 5Aza for 48 h were co-cultured with MC38G cells (1:5) for 6 h, and then cancer cell phagocytosis by macrophages were analyzed by flow cytometry.

C, Fold changes of MMP mRNA levels in DMSO or 5Aza-treated PMs according to mRNA sequencing assays.

All data were analyzed with Student’s t test. (n.s., not significant)

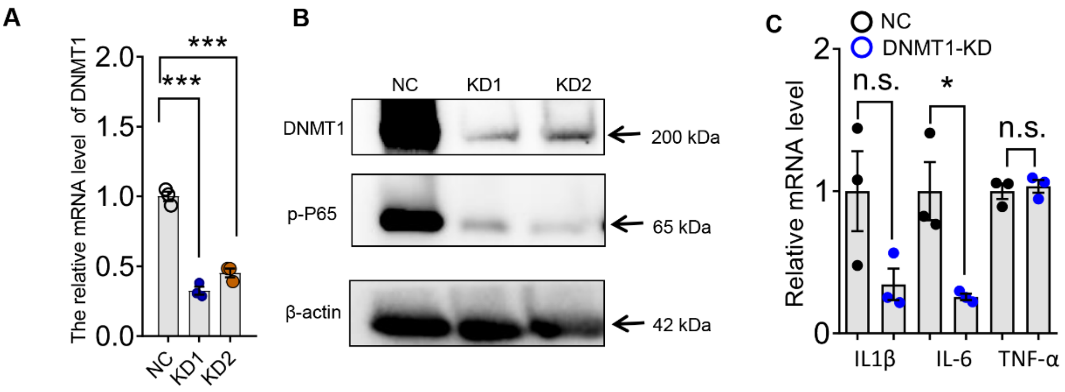

Figure S7. DNMT1 silence attenuates p65 phosphorylation.

A, mRNA levels of DNMT1 in Raw 264.7 cells transfected with a control shRNA (NC) or DNMT1-specific shRNA (KD1 and KD2).

B, Immunoblotting assays of DNMT1 and p-p65 in Raw 264.7 cells described in **A**.

C, mRNA levels of cytokines in Raw 264.7 cells transfected with NC or KD1.

All data were analyzed with Student's t test. (***P < 0.005)

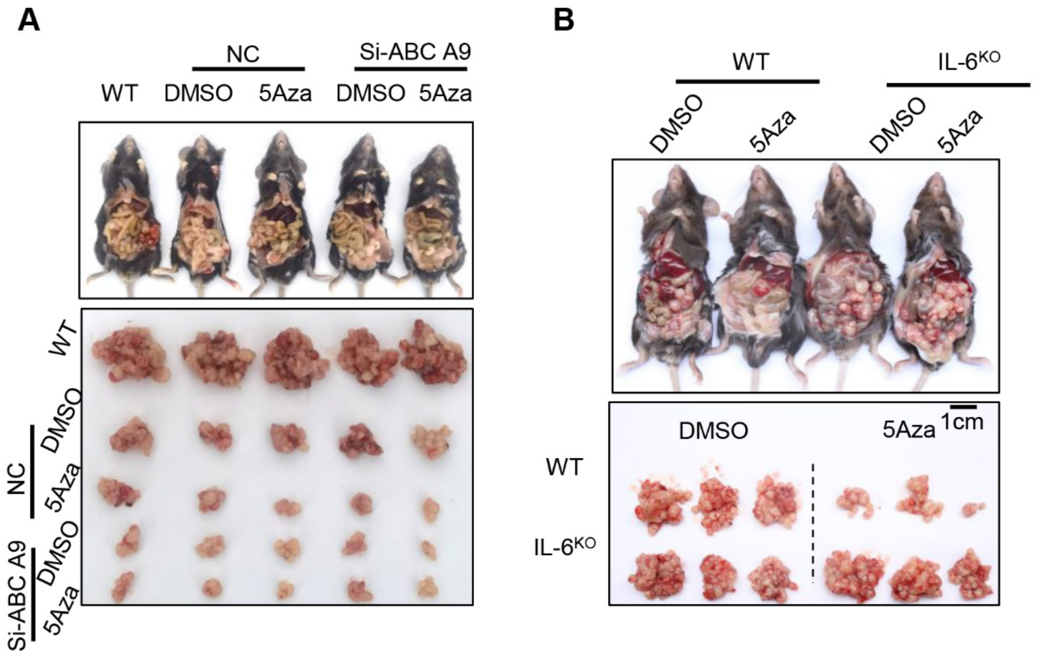

Figure S8. 5Aza inhibits CRC-PC suppression through ABC A9 and IL-6.

A, Tumor nodes of MC38 cells from the six-week-old male WT mice treated with Si-NC/ABC A9 BMDMs. Tumor-seeded models were established and treated as described in **Figure S1A** on day 0 and the tumor nodules were observed on day 14. Representative result was shown.

B, Six-week-old male WT or IL-6^{KO} mice were intraperitoneally inoculated with MC38 cells (1.0×10^6 cells in 100 μL PBS per mouse) on day 0 and then administered with 5Aza on days 0, 2, and 4. Tumor nodules were observed on day 14. Representative result was shown. (n = 3)

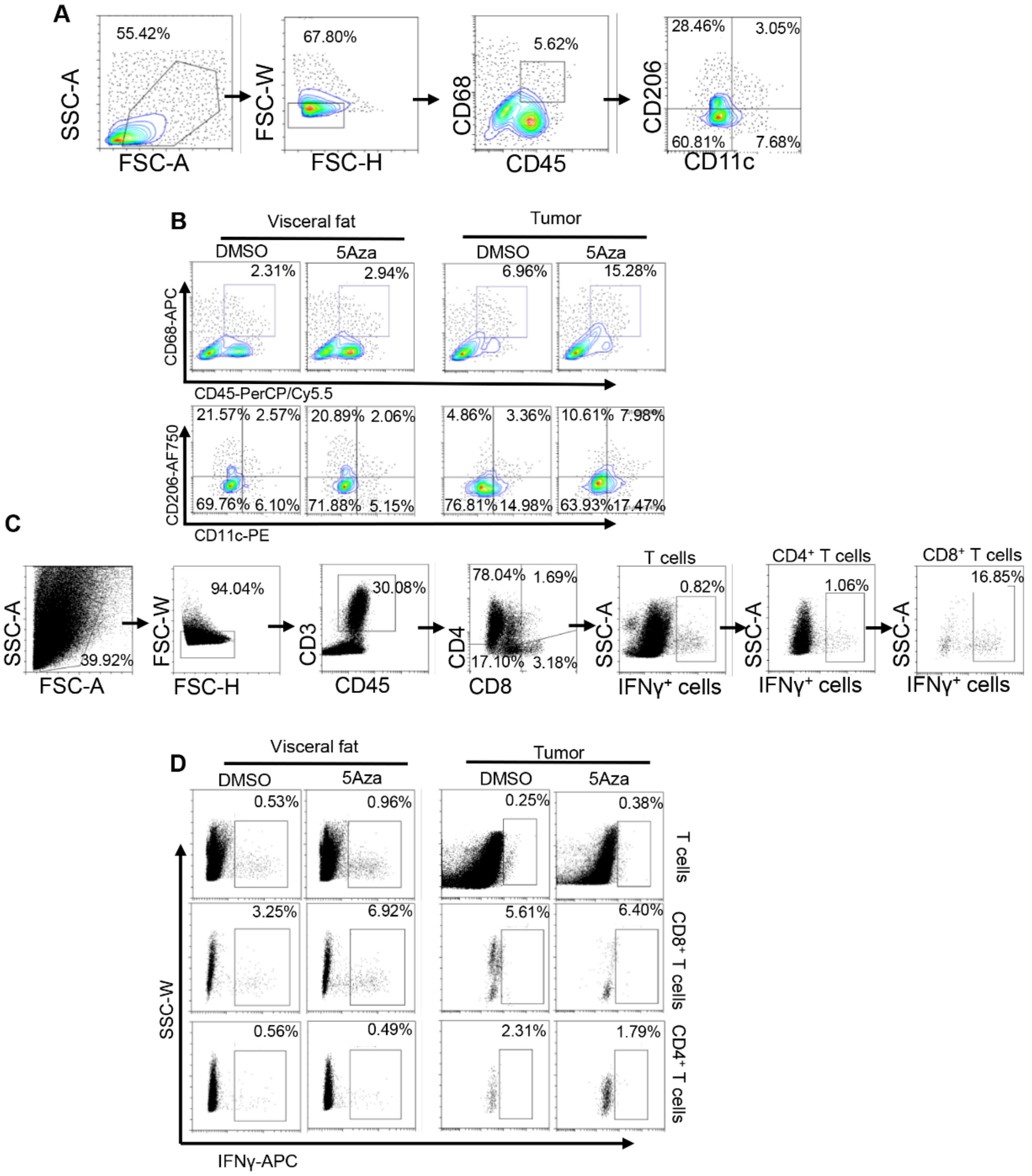

Figure S9. 5Aza synergizes chemotherapy of CRC-PC in mice and regulates activity of stromal macrophages and T cells in CRC patients

A, FACS gating strategy for total macrophages, M1-like and M2-like macrophages from human tissues. Debris and doublets were removed, and human macrophages were then assessed as $CD45^{+}CD68^{+}$. M1-like macrophages were marked as $CD45^{+}CD68^{+}CD11c^{+}CD206^{-}$. M2-like macrophages were assessed as $CD45^{+}CD68^{+}CD11c^{-}CD206^{+}$.

B, Frequencies of stromal macrophages and the subpopulations in omental fats and CRC tissues from the patients. Stromal cells of visceral fats and CRC tissues were collected and stimulated by DMSO or 5Aza for 48 h. Then macrophages and the subpopulations were analyzed by flow cytometry. Representative result was shown. (n = 3)

C, FACS gating strategy for T cells, $CD4^+$ T cells and $CD8^+$ T cells in human tissues. Debris and doublets were removed, and $IFN\gamma^+$ T cells were then assessed as $CD45^+CD3^+IFN\gamma^+$, $IFN\gamma^+CD4^+$ T cells were marked as $CD45^+CD3^+CD4^+CD8^-IFN\gamma^+$, $IFN\gamma^+CD8^+$ T cells were assessed as $CD45^+CD3^+CD4^-CD8^+IFN\gamma^+$.

D, Percentage of $IFN\gamma^+$ T cells in the stromal cells of visceral fats and CRC tissues as described above in **B**. Representative result was shown. (n = 3)
